# EprX associates with concurrent shifts in antimicrobial resistance and virulence in clinical bloodstream *E. coli*: a putative adaptive node for bacterial fitness

**DOI:** 10.3389/fcimb.2026.1919001

**Published:** 2026-08-26

**Authors:** Afeng Wang, Yingying Guo, Jichao Zhu, Yuliang Sun, Zhaochen Huang, Tinliang Lin, Chen Liang, Cunnan Xu, Xinhua Qiang, Jie He, Hongchang Zhou, Longwen Shu, Xiang Wang, Yibin Lin

**Affiliations:** 1 School of Medicine, Huzhou Normal University, Huzhou, China; 2 School of Life, Huzhou Normal University, Huzhou, Zhejiang, China; 3 Clinical Laboratory, Huzhou Central Hospital, Huzhou, Zhejiang, China; 4 School of Science, Huzhou Normal University, Huzhou, Zhejiang, China; 5 Clinical Laboratory, First People’s Hospital Affiliated to Huzhou Normal University, Huzhou Normal University, Huzhou, Zhejiang, China; 6 Infection Disease Department, First People’s Hospital Affiliated to Huzhou Normal University, Huzhou Normal University, Huzhou, Zhejiang, China; 7 Key Laboratory of Vector Biology and Pathogen Control of Zhejiang Province, Huzhou Normal University, Huzhou, Zhejiang, China; 8 Zhejiang Key Laboratory of Zero Magnetic Medicine, Affiliated Hangzhou First People’s Hospital, School of Medicine, Westlake University, Hangzhou, Zhejiang, China

**Keywords:** antibiotic resistance, bloodstream infections, *E. coli*, pentapeptide repeat proteins (PRPs), virulence

## Abstract

Bloodstream infections (BSIs) caused by *E. coli* represent a growing global threat, driven by escalating antimicrobial resistance (AMR) and sustained virulence. However, the regulatory mechanisms linking these two phenotypes remain poorly understood. Here, we identify *EprX*, a previously uncharacterized YjbI-type pentapeptide repeat protein (PRP), a locus that our data suggest may influence metabolic and transcriptional profiles in clinical BSI *E. coli* isolates. Genomic screening of 85 clinical BSI strains reveals that *eprX* is present in 21.2% of isolates, often within distinct genomic contexts suggestive of mobile acquisition. Using λ-Red recombineering, we constructed *eprX* knockout mutants. Loss of *eprX* is associated with altered antimicrobial resistance profiles, increasing susceptibility to gentamicin, ciprofloxacin, and levofloxacin. This phenotype is consistent with upregulation of outer membrane porin genes (*ompC*, *ompF*) and downregulation of multidrug efflux pump genes (*macB*, *mdtC*, *emrB*) and two-component regulatory system genes. *eprX* deficiency also appears to correlate with attenuated virulence in our assays, as evidenced by improved survival of *Galleria mellonella* larvae (65–95% at 72 h post-infection vs. 40–60% for wild-type strains) and reduced adhesion to and invasion of human HeLa cells. Transcriptomic profiling reveals that *eprX* carriage is associated with broad, coordinated shifts in the expression of genes involved in LPS transport (*lptG*/*lptF*), type ;II secretion system components (*gspD*/*gspE*/*gspF*), autotransporter adhesins (*ag43*), and flagellar assembly, suggesting potential disruptions in outer-membrane integrity, biofilm formation, and virulence programs. Our data suggests that *eprX* is a genetic locus whose presence correlates with concurrent shifts in resistance maintenance and virulence traits, representing a putative adaptive node within the *E. coli* fitness landscape.

## Introduction

*E. coli* bacteremia is among the most frequent and clinically critical bloodstream infections (BSIs) worldwide. It poses major therapeutic challenges due to its high epidemiological burden and the escalating threat of antimicrobial resistance (AMR) (Bonten et al., 2021; MacKinnon et al., 2021). As the leading causative pathogen across various healthcare settings, *E. coli* accounts for roughly 15–20% of monomicrobial BSIs and primarily originates from the gastrointestinal tract, with urosepsis being the most common source (Diekema et al., 2019; Rodriguez et al., 2021). It affects both community-onset and healthcare-associated cases, particularly impacting vulnerable populations such as the elderly and immunocompromised. This places a substantial strain on healthcare systems through prolonged hospitalization and high ICU admission rates (King et al., 2024; Kosa et al., 2025).

However, managing this prevalent infection is increasingly complicated by AMR. Notably, the endemic production of extended-spectrum β-lactamases (ESBLs) confers resistance to critical third-generation cephalosporins. Compounding this threat are carbapenem-resistant *E. coli* (CRE) and high global rates of fluoroquinolone resistance (Ng et al., 2025; Ramatla et al., 2023). These resistance patterns severely limit empirical treatment options, leading to higher rates of delayed effective therapy—a factor directly correlated with increased mortality. They also force greater reliance on last-line agents such as polymyxins or novel β-lactam/β-lactamase inhibitor combinations, which carry significant toxicity risks (Aljohni et al., 2025; Bidell et al., 2016; Walker et al., 2022). Consequently, addressing this dual challenge requires robust antimicrobial stewardship, rapid diagnostic advancements (e.g., MALDI-TOF MS), and continuous surveillance to guide effective therapy and curb the rising tide of multidrug-resistant infections.

Pentapeptide repeat proteins (PRPs) constitute a phylogenetically widespread and structurally heterogeneous superfamily. Unlike many protein families defined by linear sequence conservation, PRPs are unified by a distinctive right-handed quadrilateral β-helix (RQBH) fold. This architecture arises from the stacking of tandem pentapeptide repeats, forming an extended, planar structure stabilized by a central hydrophobic core (Bateman et al., 1998; Hegde et al., 2005; Xu and Kennedy, 2020). Although the structural framework of PRPs is well established, their precise physiological functions—particularly within pathogenic bacteria—remain an active frontier of research, revealing significant functional pleiotropy across taxa.

Functionally, PRPs participate in diverse cellular processes ranging from membrane transport to antimicrobial resistance. For instance, while specific PRPs such as RfrA may contribute to the acquisition of essential metal ions, others like Qnr and MfpA are associated with elevated baseline fluoroquinolone tolerance by acting as DNA mimics. These proteins competitively inhibit quinolone binding by obstructing the interaction between DNA gyrase and DNA (Feng et al., 2021; Zhang and Kennedy, 2021).

Beyond resistance mechanisms, the synaptic vesicle glycoprotein 2C lumenal domain (SV2C-LD) harbors a PRP domain that functions as a receptor for botulinum neurotoxin A (BoNT/A) (Dong et al., 2006; Zhang and Kennedy, 2021). In this context, the PRP motif mediates toxin binding through β-strand complementarity, extending intermolecular β-sheet architecture to secure high-affinity interactions within the vesicle lumen (Benoit et al., 2014; Yao et al., 2016). Beyond its role in neurotoxin recognition, the PRP domain also serves as a structural scaffold for certain bacterial ubiquitin E3 ligases. For example, the N-terminal regions of *Salmonella* SopA and *E. coli* NleL both contain PRP motifs that, while not directly catalyzing ubiquitination, facilitate critical protein–protein interactions with host factors such as tripartite motif (TRIM) family E3 ligases or E2 conjugating enzymes (Lin et al., 2011; Zhang and Kennedy, 2021; Zhang et al., 2006). By acting as molecular platforms, these PRP-containing domains enable pathogenic bacteria to manipulate host ubiquitination pathways, thereby subverting immune defenses and promoting infection (Zhang and Kennedy, 2021).

Additionally, PRPs assume non-enzymatic roles as specialized chaperones—such as *Bacillus subtilis* PrsA, which assists in the maturation of extracellular enzymes—or function as structural scaffolds that preserve cell envelope integrity and form rigid spore coats. These findings underscore that PRPs are not merely hypothetical open reading frames but integral components of fundamental metabolic and physiological pathways.

Despite being prevalent in the genomes of major Gram-negative pathogens such as *Pseudomonas aeruginosa* and *Vibrio cholerae*, the contribution of PRPs to bacterial virulence remains critically underexplored (Jacoby and Hooper, 2013). Emerging evidence, however, suggests several key links to pathogenicity. SopA is a multidomain protein containing a PRP domain and a HECT-type E3 ligase. Its N-terminal PRP domain suppresses host immune responses by interfering with the host TRIM56/TRIM65 RING E3 ubiquitin ligases, thereby enhancing the infectivity and pathogenicity of *Salmonella enterica* serovar Typhimurium (Zhang and Kennedy, 2021). PPE10 contains a pentapeptide repeat domain (MPTR subfamily) and is essential for capsule integrity and virulence in pathogenic mycobacteria (e.g., *Mycobacterium marinum*), affecting phenotypes related to surface localization and phagosomal escape (Ates et al., 2016). The secreted YjbI-type PRP appears to correlate with altered susceptibility of Gram-negative bacteria to antibiotics of varying molecular size, acting through​ changes in outer-membrane composition and stress-response pathways, and may thereby contribute to environmental adaptation and survival (Costafrolaz et al., 2026). Additionally, due to their structural properties, PRPs may serve as scaffolds or accessory components for type V and VI secretion systems (T5SS/T6SS), aiding in the delivery of effector toxins (Lennings et al., 2018).

While the structural conservation of PRPs is well established, a significant knowledge gap remains regarding their precise function in disease. This highlights an urgent need for future research—particularly phenotypic analyses of PRP deletion mutants in clinical isolates—to validate their potential as novel virulence factors and therapeutic targets.

Through analysis of the genomes of 85 *E. coli* isolates from clinical bloodstream infections, we identified a previously unreported PRP, which we named EprX. This protein is prevalent among clinical bloodstream isolates of *E. coli* and shows a significant association with specific antimicrobial resistance phenotypes. Based on our phenotypic observations, we hypothesize that *eprX* carriage is associated with measurable shifts in global transcriptional profiles and metabolic output. In this tentative working model, the presence of *eprX* may correlate with a distinct allocation of cellular resources between antibiotic tolerance and virulence factor production, although direct regulatory mechanisms remain to be established.

To test this hypothesis, this study aims to: (i) elucidate the phylogenetic distribution and genetic context of EprX among clinical *E. coli* strains; (ii) characterize the impact of EprX expression on correlations between EprX status and antibiotic susceptibility and both *in vitro* and *in vivo* virulence phenotypes; and (iii) employ transcriptomic profiling to dissect the underlying molecular networks that appear to shift in association with loss of EprX. Our findings reveal a novel association wherein a single protein locus correlates with the concurrent shaping of the antimicrobial resistance landscape and virulence potential of bloodstream *E. coli*.

## Materials and methods

### Bacterial strains and clinical isolates

A total of 85 non-duplicate *E. coli* isolates were collected from clinical blood specimens at Huzhou Central Hospital (Huzhou, China) between January and December 2023 (**Supplementary Table 1**). Isolates were recovered through routine diagnostic workflows for BSIs and identified using standard microbiological methods, including colony morphology, Gram staining, and matrix-assisted laser desorption/ionization time-of-flight mass spectrometry (MALDI-TOF MS). For ambiguous cases, species identity was confirmed by sequencing the 16S rRNA gene.

### Identification of the eprX Gene via PCR and Sanger sequencing

The eprX gene was identified via PCR amplification, Sanger sequencing, and bioinformatic analysis. Initially, genomic DNA served as the template for PCR amplification, utilizing a specifically designed primer pair to target the region of interest, using primer pairs: 5’-CTGGAAACGCCGTATAAACCGCATCC-3’ and 5’-CGGGCAAATGACCGCCGATATCGAAG-3’. The PCR reaction mixture was composed of standard components, including high-fidelity DNA polymerase, deoxynucleotide triphosphates (dNTPs), an appropriate reaction buffer, MgCl_2_, the template DNA, and the designated primers, with the thermal cycling conditions carefully optimized for parameters such as annealing temperature and extension time based on the primer specifications and the expected amplicon length. Following the amplification phase, the PCR products were subjected to agarose gel electrophoresis to verify the presence of a single DNA fragment corresponding to the anticipated size, after which the target DNA band was excised from the gel and purified using a commercial gel extraction kit. The resulting purified PCR product was then forwarded for first-generation Sanger sequencing, employing the original forward and reverse primers to acquire comprehensive double-stranded sequence data (**Supplementary Table 1**). This finalized consensus sequence underwent rigorous Open Reading Frame (ORF) analysis, where bioinformatics tools (including NCBI ORF Finder)​ were utilized to scan all potential reading frames and pinpoint the longest ORF corresponding to the EprX protein, thereby designating its nucleotide sequence as the definitive *eprX* gene.

### Genetic manipulation

The *eprX* gene knockout strain was constructed in *E. coli* utilizing the λ-Red recombineering system in accordance with a homologous recombination strategy (Datsenko and Wanner, 2000). Initially, template DNA for the knockout cassette was derived from the pKD3 plasmid, which contains a chloramphenicol resistance marker (*cat*). Subsequently, a primer pair was designed to amplify this resistance cassette, incorporating 40–50 bp homologous arms specific to the upstream and downstream regions of the target PRP gene to facilitate subsequent homologous recombination. Specifically, the forward primer (TAATACTCCCTAAAACTGTCTTATTTAGTCAACGTAAGAGGATTACAATCGGCAATAGTTACCCTTATTATCAAG) and reverse primer (GATATTTCAAGGTGTAACTTTGTCACTCATAATTCAATGACACTGAAATATCGGGAGCTGTAATATAAAAACCTTCTTC) were employed in PCR amplification using pKD3 as the template, yielding a linear DNA fragment composed of the cat gene flanked by homologous sequences corresponding to the *eprX* gene locus. Following amplification, the purified PCR product was introduced into electrocompetent *E. coli* cells via electroporation under standard conditions. These cells harbored the λ-Red recombineering machinery expressed from a temperature-sensitive plasmid pKD46. After electroporation, the cells were immediately subjected to recovery in SOC medium at 30°C to enable expression of the λ-Red proteins (Exo, Bet, Gam), which orchestrate homologous recombination. During this recovery phase, the linear DNA fragment recombined with the chromosomal *eprX* locus through its flanking homologous arms, thereby replacing the native *eprX* gene with the chloramphenicol resistance cassette. Subsequently, the cells were plated onto LB agar plates supplemented with chloramphenicol (25 µg/mL) and incubated overnight at 37°C to select for recombinant colonies that had successfully undergone allelic replacement. Finally, putative knockout colonies were verified by colony PCR using primers annealing outside the homologous recombination arms to confirm precise integration of the *cat* gene and deletion of the *eprX* gene, with final confirmation achieved through DNA sequencing of the amplified product.

### Antimicrobial susceptibility testing

The bacterial isolate was initially subcultured on a non-selective agar medium, specifically Luria Broth Agar (LBA), and incubated at 35 ± 2°C for 18–24 hours to confirm purity and achieve the optimal growth phase. Following this, several isolated colonies were transferred into a tube containing sterile saline or broth, and the turbidity of the resulting suspension was adjusted to match the 0.5 McFarland standard, corresponding to approximately 1.5 × 10^8^ CFU/mL. Antimicrobial powders sourced from certified manufacturers were dissolved in appropriate solvents, such as​ sterile distilled water, dimethyl sulfoxide (DMSO), or phosphate-buffered saline (PBS), according to the manufacturer’s instructions to prepare stock solutions, which were subsequently stored at –70°C or below to preserve stability. A two-fold serial dilution technique was then performed in 96-well microtiter plates by diluting the stock solution in cation-adjusted Mueller-Hinton Broth (CAMHB) to establish a concentration gradient typically ranging from 0.125 µg/mL to 256 µg/mL, with each well containing 100 µL of the antimicrobial dilution alongside designated sterility and growth control wells. Subsequently, 100 µL of the standardized bacterial inoculum, pre-diluted 1:100 in CAMHB, was dispensed into all test wells using a multi-channel pipette, yielding a final inoculum density of approximately 5 × 10^5^ CFU/mL per well. The sealed microtiter plates were incubated aerobically at 35 ± 2°C for 16–20 hours. Post-incubation, the Minimum Inhibitory Concentration (MIC) was determined visually by identifying the lowest antibiotic concentration exhibiting no visible turbidity relative to the growth control well. Quality control was ensured by concurrently testing CLSI (Clinical and Laboratory Standards Institute)-recommended reference strains, including *E. coli* ATCC 25922 to validate assay accuracy and reproducibility. Finally, the acquired MIC values were interpreted utilizing the CLSI M100 (Standard document) to classify isolates as Susceptible (S), Intermediate (I), or Resistant (R), and statistical analyses were conducted using GraphPad Prism software (version 9).

### Galleria mellonella infection model

*Galleria mellonella* (greater wax moth) larvae were obtained from Sichuan Mingdiao Workshop Fishing Tackle Co., Ltd., China, with only healthy, actively moving larvae in the final instar stage selected for experimentation to minimize variability. Larvae exhibiting visible melanization, lethargy, molting, or a body weight outside the range of 250–350 mg were excluded from the study. Prior to inoculation, larvae were randomly assigned to experimental groups (sham-infected control, wild-type-infected, *ΔeprX*-infected) using a computer-generated randomization list, then briefly surface-sterilized by immersion in 70% ethanol for 30 seconds, followed by two washes in sterile phosphate-buffered saline (PBS) to remove residual ethanol and potential surface contaminants. After drying on sterile filter paper and a 1-hour acclimation at room temperature, infections were established via last proleg injection using a Hamilton syringe fitted with a 30-gauge needle, a route designed to mimic natural bacterial dissemination, with each experimental group inoculated with a standardized inoculum of 1×10^6^ CFU in 5 μL of sterile PBS; control groups received an equivalent volume of sterile PBS alone (sham-injected). The injection site was swabbed with 70% ethanol before and after inoculation to prevent wound infection. Post-injection, larvae were immediately transferred to sterile Petri dishes lined with moistened filter paper to maintain humidity and incubated at a constant temperature of 37°C, reflecting mammalian physiological temperature, under static conditions to simulate human systemic infection, with a subset of control groups maintained at 30°C, optimal for insect physiology, to confirm that mortality was due to infection rather than temperature stress. Larval survival was monitored every 12 hours over a total period of 72 hours by blinded investigators (Petri dishes were coded, with group identities concealed until the endpoint), with larvae deemed dead upon showing no response to tactile stimulation alongside visible melanization or tissue degradation. Kaplan–Meier survival curves were generated to visualize survival probabilities over time, and statistical differences in survival between groups were assessed using the Log-rank (Mantel–Cox) test. All statistical analyses were performed using GraphPad Prism v9.0, with a *p*-value of less than 0.05 considered statistically significant, and data presented as Kaplan–Meier plots with 95% confidence intervals.

### Cell culture and infection assays

Cell lines and culture conditions. This study utilized a human-derived epithelial cell line: HeLa (ATCC CCL-2), which is a cervical adenocarcinoma cell line. The cell line was maintained in high-glucose Dulbecco’s Modified Eagle Medium (DMEM) supplemented with 10% (v/v) heat-inactivated fetal bovine serum (FBS), 100 U/mL penicillin, and 100 µg/mL streptomycin. Cells were incubated at 37°C in a humidified atmosphere containing 5% CO_2_, and for all infection and adhesion/invasion assays, cells were seeded into appropriate plates (e.g., 24-well or 96-well plates) and used upon reaching 80 – 90% confluency.

Bacterial strains and growth conditions. The bacterial strain used in this study was listed in **Supplementary Table 1**. Bacteria were cultured overnight in Lysogeny Broth (LB) medium at 37°C with shaking at 180 rpm. Prior to infection assays, bacterial cultures were harvested by centrifugation (5,000 × g, 10 min), washed twice with sterile PBS, and resuspended in DMEM without antibiotics, while the optical density at 600 nm (OD_600_) was measured to standardize the inoculum concentration.

Adhesion assay. To quantify bacterial adhesion to host cells, a procedure was performed beginning with the preparation of monolayers of HeLa cells seeded in 24-well plates and grown to confluence as described previously (Wang et al., 2022). During the infection step, the culture medium was removed, and cells were washed once with PBS before adding one milliliter of bacterial suspensions prepared in DMEM (without antibiotics) to achieve a multiplicity of infection (MOI) of 100:1. Plates were then centrifuged at 800 × g for 5 minutes at room temperature to synchronize bacterial contact with host cells, followed by incubation at 37°C with 5% CO_2_ for 1 hour to allow for adhesion. Subsequently, the supernatant was removed, and wells were vigorously washed three times with sterile PBS to remove non-adherent bacteria. To release adherent bacteria, cells were lysed with 0.1% (v/v) Triton X-100 in PBS for 10 minutes at room temperature. Finally, serial dilutions of the lysate were plated onto LB agar plates and incubated overnight at 37°C to count colony-forming units (CFU) per well, which was expressed as CFU per 10^6^ cells.

Invasion assay. The invasion assay, designed to measure the ability of bacteria to penetrate and survive within host cells, was conducted using the Kanamycin Protection Assay to differentiate between intracellular and extracellular bacteria (Sharma and Puhar, 2019). Host cells (HeLa) were seeded in 24-well plates and infected as described in the Adhesion Assay. Following the 1-hour adhesion/entry period, the medium was replaced with fresh DMEM containing kanamycin (typically 50 – 100 µg/mL) to kill extracellular and surface-bound bacteria, and plates were incubated for an additional 1–2 hours. After kanamycin treatment, cells were washed three times with PBS to remove residual antibiotic, and intracellular bacteria were released by lysis with 0.1% Triton X-100. Lysates were then serially diluted, plated on LB agar, and incubated overnight, with the number of resulting colonies counted and reported as CFU per well to represent the invasive capacity of the bacteria.

### RNA sequencing and data analysis

RNA sequencing and data analysis were performed as described previously (Gao et al., 2026; Mao et al., 2019). Total RNA was extracted from three independent biological replicates per strain​ (ECO1, ECO30, ECO42 and their matched *ΔeprX* mutants) using the RNeasy Mini Kit (Qiagen, MD, USA), with quality assessed via agarose gel electrophoresis, NanoPhotometer spectrophotometry (IMPLEN, CA, USA), Qubit^®^ 2.0 Fluorometer (Life Technologies, CA, USA), and Bioanalyzer 2100 system (Agilent Technologies, CA, USA). All samples met pre-sequencing quality thresholds: RNA Integrity Number (RIN) >8.0, 28S/18S rRNA ratio >1.8, and no detectable genomic DNA contamination. Sequencing libraries were constructed from 3 μg RNA per sample using the NEBNext Ultra RNA Library Prep Kit (NEB, MA, USA) following poly-A selection with magnetic beads. Briefly, mRNA was fragmented, followed by first- and second-strand cDNA synthesis. After end-repair, A-tailing, and adapter ligation, cDNA fragments (150–200 bp) were purified using the AMPure XP system (Beckman Coulter, IN, USA). Libraries were amplified via PCR, validated on a Bioanalyzer 2100 (Agilent, CA, USA), and sequenced on an Illumina HiSeq 2000 platform (100 bp paired-end reads) after cluster generation on a cBot system (Illumia, CA, USA). Raw reads were quality-filtered using Trimmomatic to remove adapters and low-quality bases, calculating Q20, Q30, and GC content (Bolger et al., 2014). Clean reads were mapped to the reference genome using Bowtie2 (v2.2.6) (Langmead and Salzberg, 2012). Gene expression levels were quantified as FPKM values using HTSeq-count (Anders et al., 2015; Mortazavi et al., 2008; Trapnell et al., 2010). Differential expression analysis was performed using DESeq2 (v1.16.1; based on the negative binomial distribution) for datasets with biological replicates (adjusted *P* < 0.05) (Benjamini and Hochberg, 1995; Love et al., 2014), or edgeR (v3.18.1) for those without replicates (adjusted *P* < 0.05 and |fold change| ≥ 2), applying Benjamini-Hochberg correction for multiple testing (McCarthy et al., 2012; Robinson et al., 2010).

### GO and KEGG enrichment analysis of differentially expressed genes

GO and KEGG enrichment analyses of differentially expressed genes were performed as described previously (Mao et al., 2019). To elucidate the biological implications of the differentially expressed genes (DEGs), we performed Gene Ontology (GO) and Kyoto Encyclopedia of Genes and Genomes (KEGG) pathway enrichment analyses. GO enrichment was conducted using the topGO package in R, employing an algorithm designed to mitigate gene length bias (Alexa et al., 2006). Statistically significant GO terms were defined as those with a false discovery rate (FDR)-adjusted *P*-value < 0.05 (Oshlack and Wakefield, 2009; Young et al., 2010). For pathway analysis, we utilized the clusterProfiler package to map DEGs onto KEGG reference pathways (Wu et al., 2021). The KEGG database serves as a comprehensive resource for interpreting the higher-order functional properties of biological systems based on genomic and molecular datasets (http://www.genome.jp/kegg/) (Kanehisa et al., 2025).

### Statistical analysis

Statistical analyses were performed using GraphPad Prism version 9.0 (GraphPad Software, San Diego, CA, USA). For comparisons between two groups, two-tailed Student’s *t*-test was applied. Survival analysis in the *Galleria mellonella* infection model was conducted using Kaplan–Meier survival curves, and statistical differences were evaluated by the Log-rank (Mantel–Cox) test. For multi-group comparisons in adhesion and invasion assays, one-way ANOVA with Tukey’s *post-hoc* test was used.

Differential expression analysis of transcriptomic data was performed using the DESeq2 R package (version 1.16.1) for samples with biological replicates, based on a negative binomial distribution model. For samples without biological replicates, the edgeR R package (version 3.18.1) was used with a scaling normalization factor. Adjusted *p*-values were calculated using Benjamini and Hochberg’s false discovery rate (FDR) method, and genes with an adjusted *P* < 0.05 and absolute fold change ≥ 2 were considered significantly differentially expressed. GO enrichment was performed using the topGO R package, and KEGG pathway enrichment was conducted using the clusterProfiler R package. In all analyses, *P <* 0.05 was considered statistically significant.

## Results

### Genomic prevalence of the eprX gene in clinical E. coli isolates

**Figure 1:**
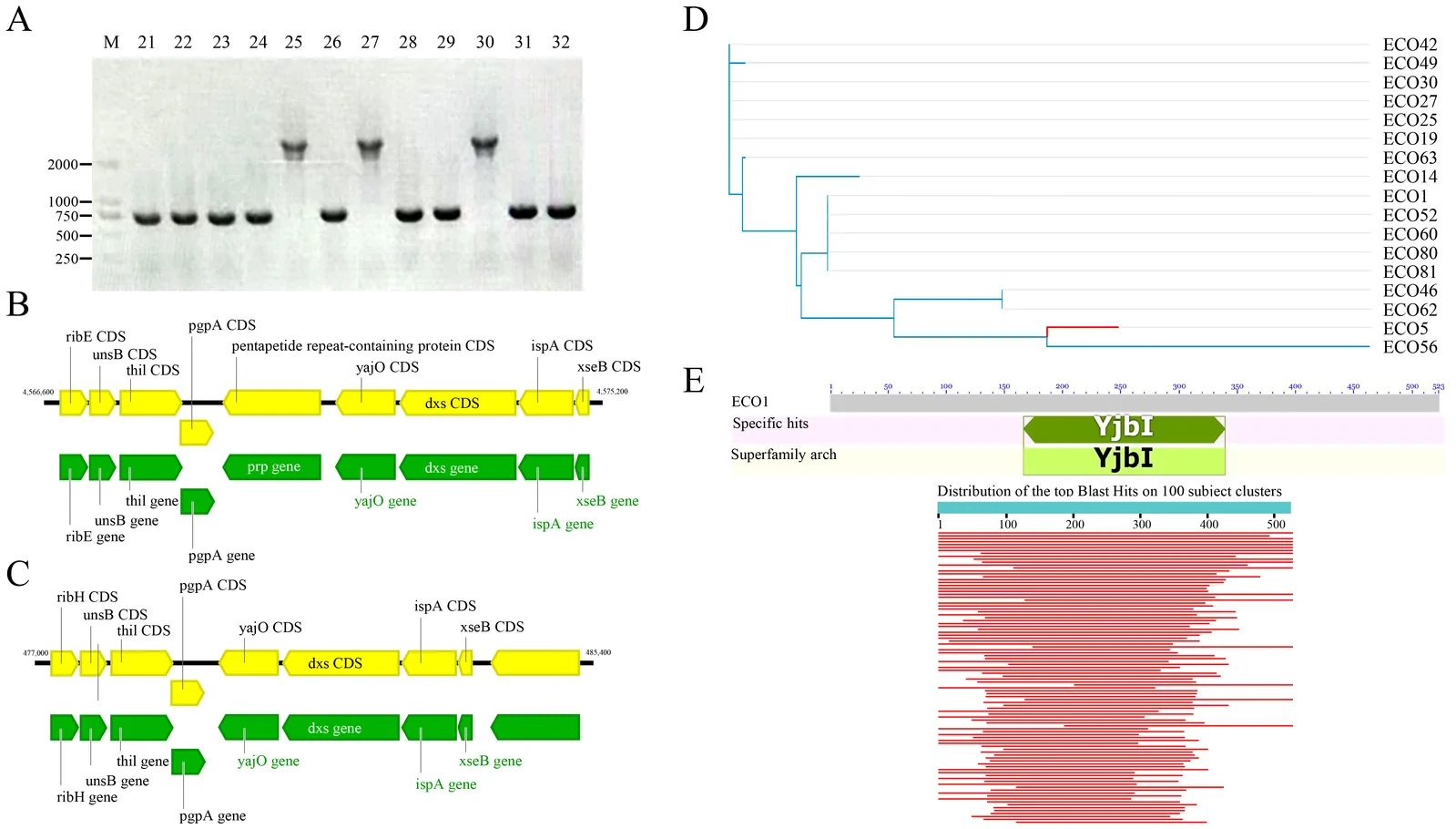
Comprehensive analysis of the *eprX* Gene in clinical *E. coli* isolates. **(A)**​ PCR detection of *eprX* in clinical *E. coli* isolates ECO21–ECO32. Agarose gel electrophoresis of the PCR products shows a ~2.3-kb band when the gene is present and a ~750-bp band when it is absent. Lane M, DNA ladder. **(B)**​ Genomic region (coordinates 4,566,600–4,575,200) of *E. coli* strain C16EC0626 (NCBI GenBank accession CP088656). Yellow boxes denote coding sequences (CDSs) and green boxes denote genes. Labeled genes include *pgpA*, *prp*, *yajO*, etc. The *eprX* gene is present at this locus. **(C)**​ Orthologous region (coordinates 477,000–485,400) of *E. coli* strain MS8320 (NCBI GenBank accession CP059339). Unlike C16EC0626, *eprX* is absent here. **(D)**​ Uncorrected neighbor-joining tree of EprX sequences from clinical *E. coli* isolates. Red branches denote negative distances. EprX from ECO1, ECO52, ECO60, ECO80, and ECO81 are identical; the same holds for ECO19, ECO25, ECO27, ECO30, and ECO42, as well as for ECO44, ECO46, and ECO62. **(E)**​ Domain architecture of YjbI from ECO1 and distribution of its top BLAST hits. Top:​ Domain architecture of YjbI showing specific hits (pink) and superfamily architecture (yellow). Bottom:​ Distribution of top BLAST hits across 100 subject-sequence clusters (red bars).

To determine the genomic prevalence of *eprX* in clinical *E. coli*, we analyzed 85 bloodstream infection (BSI) isolates. PCR screening detected 18 *eprX*-positive isolates (21.2%; 18/85; **Figure 1A**; **Supplementary Figure 1**).

Genomic analysis of these isolates and reference sequences from GenBank showed variation in the genomic contexts for *eprX*. In representative strain C16EC0626 (accession CP088656), *eprX* is inserted between *pgpA* and *yaiO* (**Figure 1B**), whereas it is absent in reference strain MS8320 (accession CP059339; **Figure 1C**). Sanger sequencing of all 18 positive isolates (**Supplementary Tables 2**, **S3**) enabled comparative sequence analysis. Deduced EprX protein sequences clustered into three distinct groups: Group I (ECO1, ECO52, ECO60, ECO80, ECO81), Group II (ECO19, ECO25, ECO27, ECO30, ECO42), and Group III (ECO44, ECO46, ECO62) (**Supplementary Figure 2**). A phylogenetic tree based on these sequences corroborated this grouping (**Figure 1D**).

The observed prevalence of *eprX* (21.2%) is consistent with a potentially recurrent feature among circulating clinical clones. This frequency bears resemblance to the established association of pentapeptide repeat proteins (PRPs)—notably the Qnr family—with quinolone susceptibility (Jacoby et al., 2014). Standard phenotypic antimicrobial susceptibility testing (AST) may not reliably detect such low-level resistance mechanisms. For instance, isolates carrying *qnr* and related determinants can exhibit MICs within the susceptible range despite an elevated risk of treatment failure (Kalinen et al., 2025). Furthermore, the discontinuous distribution of *eprX* in the genome is consistent with acquisition via horizontal gene transfer, a recognized mechanism for spreading resistance and virulence traits in healthcare settings. Finally, the identification of three conserved EprX variants provides a framework for tracking clonal dissemination and evaluating population-level impacts.

Sequence alignment against the NCBI database revealed that EprX contains a conserved YjbI-type pentapeptide repeat motif (**Figure 1E**), supporting its annotation as a member of the PRP family. PRPs are defined by tandem arrays of a five-amino-acid consensus motif ([S,T,A,V][D,N][L,F][S,T,R][G]) that fold into a distinctive right-handed quadrilateral β-helix (Vetting et al., 2006). Consistent with this, structural modeling using AlphaFold DB (Varadi et al., 2022) predicted that EprX is likely to adopt a helical fold stabilized by pentapeptide repeats (**Supplementary Figure 3**). This structural similarity to the YjbI family may have implications for its cellular localization and protein interactions.

### Deletion of the eprX gene is associated with increased susceptibility to multiple antibiotics

**Figure 2:**
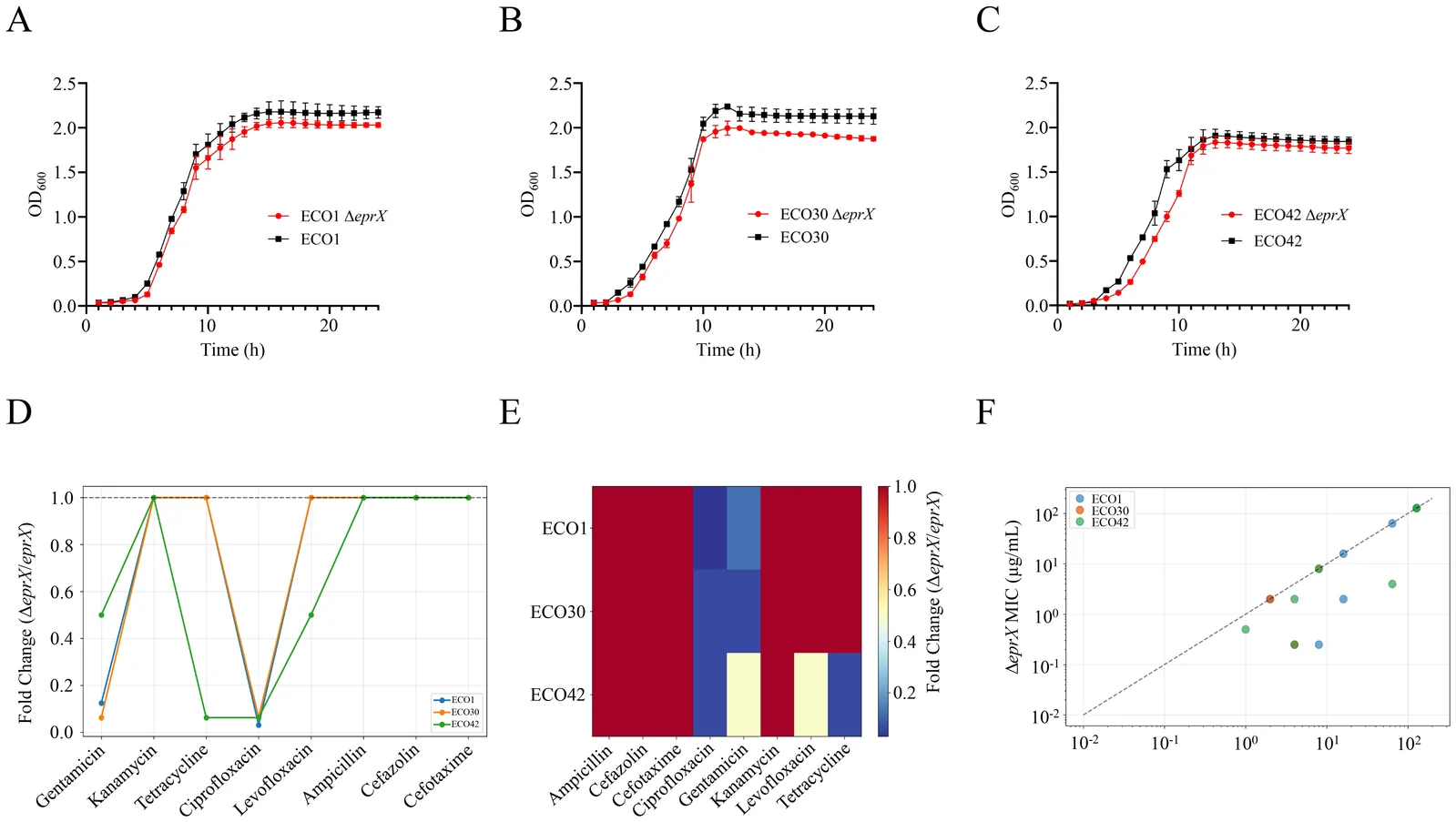
Deletion of the *eprX* gene is associated with increased susceptibility to multiple antibiotics.​ **(A–C)**​ Growth curves of *E. coli* strains ECO1, ECO30, and ECO42, measured as OD_600_ over time. Red circles and black squares represent Δ*eprX* knockout and wild-type (WT) strains, respectively. **(D)**​ Fold changes in antimicrobial resistance (Δ*eprX*/WT) for eight tested antibiotics: gentamicin, kanamycin, tetracycline, ciprofloxacin, levofloxacin, ampicillin, cefazolin, and cefotaxime. Values < 1 indicate increased susceptibility following *eprX* deletion. **(E)**​ Heatmap of fold changes (Δ*eprX*/WT) across all strains and antibiotics. Color intensity reflects the magnitude of change (red = low susceptibility, blue = high susceptibility). **(F)**​ Correlation between WT and Δ*eprX* MIC values across strains. The dashed line indicates a 1:1 relationship, with points below the line representing reduced MIC values (i.e., increased susceptibility) in the knockout strains. Data are presented as mean ± SD (n = 3 biological replicates).

To examine the potential contribution of *eprX* to antibiotic resistance, we generated *eprX* knockout mutants (Δ*eprX*) derived from three clinical *E. coli* isolates: ECO1, ECO30, and ECO42. Growth curve analyses (**Figures 2A–C**) reveal no substantial differences in growth kinetics between Δ*eprX* strains and their isogenic wild-type (WT) counterparts. All strains exhibit similar growth rates and reach comparable optical densities (OD_600_) after 24 h, indicating that *eprX* deletion does not markedly impair bacterial viability or proliferation under these experimental conditions.

We subsequently evaluated antibiotic susceptibility using standard assays (**Table 1**) and calculated fold changes in MIC values (**Figures 2D–F**). In ECO1Δ*eprX*, the MICs for gentamicin and ciprofloxacin decrease substantially—from 16 to 2 μg/mL and from 8 to 0.25 μg/mL, respectively—effectively shifting these phenotypes from resistant to susceptible. MICs for ampicillin, cefazolin, cefotaxime, tetracycline, and kanamycin remain largely unchanged. ECO30Δ*eprX* exhibits a similar profile, with reduced MICs for gentamicin (from 4 μg/mL to 0.25 μg/mL) and ciprofloxacin (to 0.25 μg/mL), while susceptibility to other agents is unaffected. In ECO42Δ*eprX*, MICs for gentamicin (4 to 2 μg/mL), ciprofloxacin (4 to 0.25 μg/mL), levofloxacin (1 to 0.5 μg/mL), and tetracycline (64 to 4 μg/mL) also decline, whereas MICs for cefazolin, cefotaxime, ampicillin, and kanamycin are stable.

**Table 1:** Results of antimicrobial susceptibility testing for different strains.

Strain	Gentamicin	Kanamycin	Tetracycline	Ciprofloxacin	Levofloxacin	Ampicillin	Cefazolin	Cefotaxime
ECO1	16(R)	16(S)	64(R)	8(R)	2(R)	128(R)	128(R)	128(R)
ECO1Δ*eprX*	2(S)	16(S)	64(R)	0.25(S)	2(R)	128(R)	128(R)	128(R)
ECO30	4(I)	8(S)	128(R)	4(R)	2(R)	128(R)	≧128(R)	128(R)
ECO30Δ*eprX*	0.25(S)	8(S)	128(R)	0.25(S)	2(R)	128(R)	128(R)	128(R)
ECO42	4(I)	8(S)	64(R)	4(R)	1(I)	128(R)	≧128(R)	128(R)
ECO42Δ*eprX*	2(S)	8(S)	4(S)	0.25(S)	0.5(S)	128(R)	≧128(R)	128(R)
ATCC25922	0.125(S)	4(S)	2(S)	0.125(S)	0.5(S)	1(S)	≦4(S)	0.5(S)
Breakpoints	≤2-≥8	≤16-≥32	≤4-≥16	≤0.25-≥1	≤0.5-≥2	≤8-≥32	≤16-≥32	≤1-≥4

Fold-change analysis (**Figures 2D–F**) quantifies these reductions, supporting a potential association between *eprX* deletion and increased susceptibility of these clinical isolates to specific antibiotics. Collectively, these phenotypic data are compatible with a possible contribution of *eprX* to modulating antimicrobial susceptibility profiles, although genetic complementation studies will be required to confirm direct causality.

### eprX deletion is associated with attenuated bacterial virulence in vivo

The role of *eprX* in pathogenesis was evaluated using a *Galleria mellonella* infection model. Survival of mutant derivatives lacking the *eprX* locus was assessed relative to their parental wild-type (WT) strains and uninfected controls over 72 hours post-infection.

**Figure 3:**
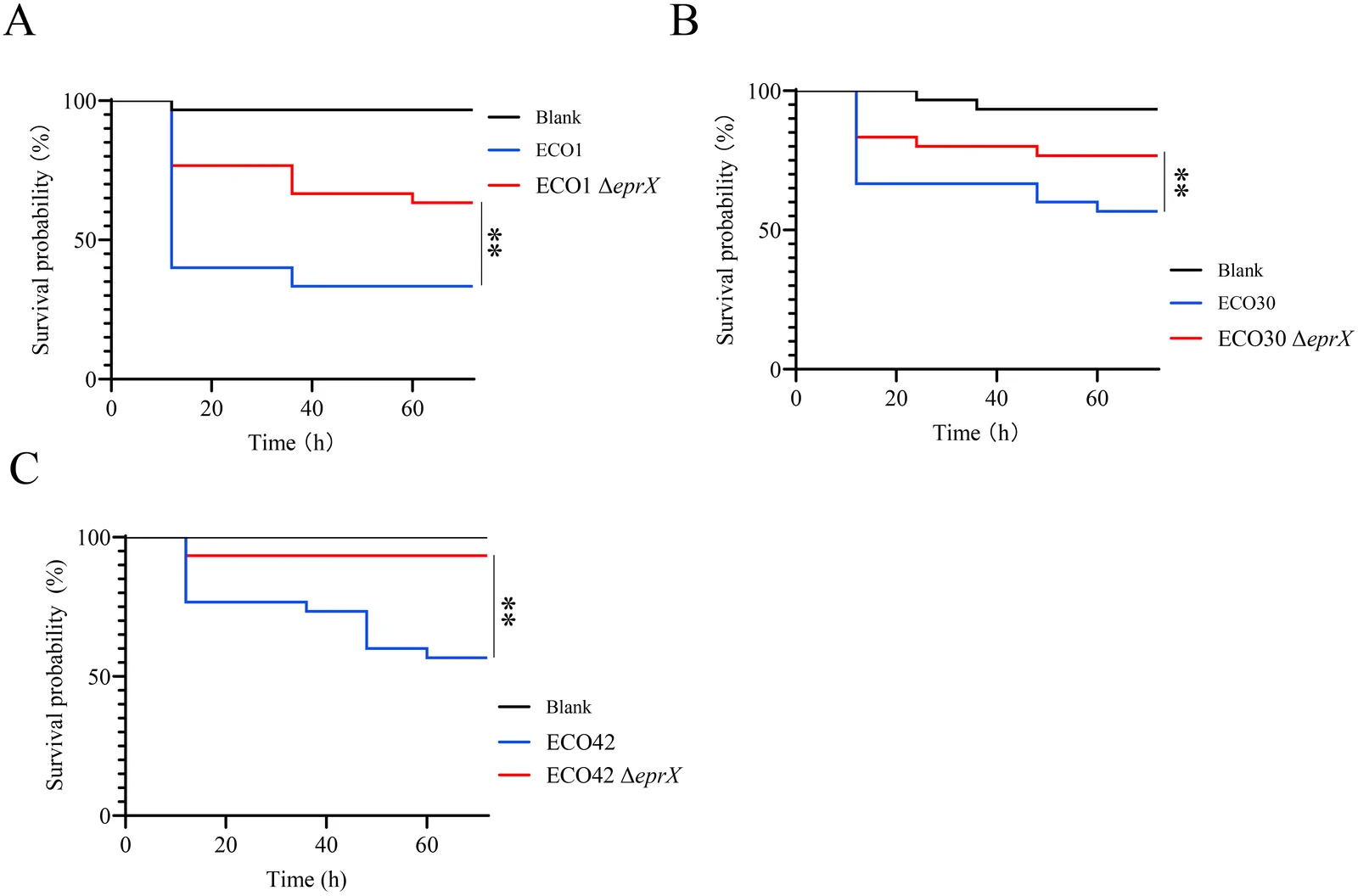
EprX deletion is linked to attenuated *E. coli* virulence in a *Galleria mellonella* infection model.​ **(A–C)** Survival curves of *Galleria mellonella* larvae infected with WT *E. coli* strains (ECO1, ECO30, and ECO42, respectively) or their isogenic Δ*eprX* mutants, alongside blank controls. In all three panels, black, blue, and red lines represent blank controls (100% survival), WT strains, and Δ*eprX* mutants, respectively. Larval survival was monitored over 72 hours post-infection. In the *Galleria mellonella* infection model, larvae infected with Δ*eprX* mutants exhibited consistently higher survival rates relative to wild-type counterparts, indicating a correlative attenuation of *in vivo* pathogenicity in strains lacking *eprX*. Data represent three independent experiments (n = 10 larvae per group per experiment). Statistical significance was determined by the log-rank (Mantel-Cox) test: *P < 0.05, **P​ < 0.01, ***P < 0.001.

Uninfected control larvae maintained 100% viability throughout the observation period (**Figure 3**). In contrast, larvae infected with WT strains (ECO1, ECO30, or ECO42) exhibited a steady decline in survival over time. By 72 hours post-infection, survival rates dropped to ~40% for ECO1 (**Figure 3A**), ~60% for ECO30 (**Figure 3B**), and ~55% for ECO42 (**Figure 3C**). Consistent with a potential contribution of *eprX* to virulence, larvae infected with the corresponding *eprX*-knockout mutants exhibited trends toward improved survival compared to their WT-infected counterparts. At 72 hours, survival reached ~65% for ECO1Δ*eprX*, ~85% for ECO30Δ*eprX*, and ~95% for ECO42Δ*eprX*. While these observations are consistent with a possible attenuation of *in vivo* virulence in the absence of *eprX*, further studies are required to establish causality.

### eprX deletion is associated with reduced adhesion and invasion in mammalian cells

To assess the effect of *eprX* loss on host cell interaction, gene-targeted knockouts were constructed in ECO1, ECO30, and ECO42. The adherence and invasion capacities of these mutants (ECO1Δ*eprX*, ECO30Δ*eprX*, and ECO42Δ*eprX*) were quantified in HeLa cells alongside wild-type (WT) controls and the ATCC 25922 reference strain.

**Figure 4:**
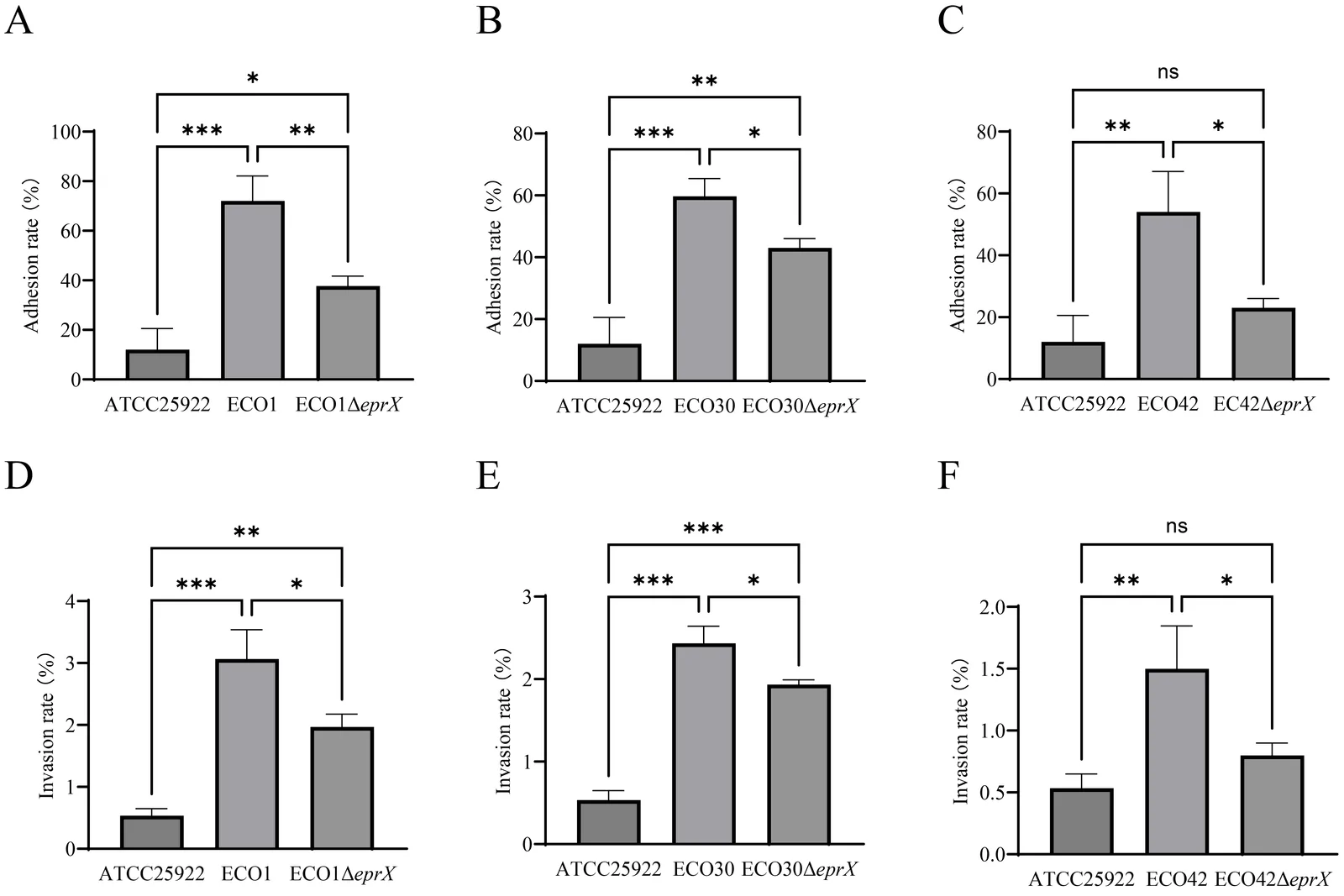
*eprX* deficiency correlates with diminished​ adhesion and invasion in mammalian cells. To evaluate the role of *eprX* in host–pathogen interactions, adhesion to and invasion of HeLa cells were assessed for three sets of *E. coli* strains: **(A, D)** ATCC25922, ECO1 (WT), and ECO1Δ*eprX***; (B, E)** ATCC25922, ECO30 (WT), and ECO30Δ*eprX*; **(C, F)** ATCC25922, ECO42 (WT), and ECO42Δ*eprX*. Panels A–C depict adhesion rates, whereas **(D–F)** depict invasion rates. Error bars represent SD from three independent experiments. Statistical significance was determined by one-way ANOVA with Tukey’s *post hoc* test (*P < 0.05, **P < 0.01, ***P < 0.001; ns, not significant).

Adhesion assays revealed that WT ECO1 attached to HeLa cells at appreciably higher levels than both ATCC25922 and its *eprX*-deficient derivative (P < 0.001 and P < 0.01, respectively; **Figure 4A**). A similar trend was observed for ECO30, in which the WT form exhibited greater adhesive ability than ATCC25922 and ECO30Δ*eprX* (P < 0.001 and P < 0.05; **Figure 4B**). In the ECO42 background (**Figure 4C**), the WT again showed increased adhesion relative to ATCC25922 (P < 0.01) and the deletion mutant (P < 0.05). However, this difference was less pronounced compared to the other lineages (P > 0.05). Thus, while a reduction in adhesion was observed in all strains upon *eprX* loss, the magnitude of this effect varied by genetic background, being most evident in the ECO1 and ECO30 contexts.

Parallel invasion experiments yielded similar results. ECO1 WT invaded cells at a significantly higher rate than ATCC25922 (P < 0.001) and its Δ*eprX* counterpart (P < 0.05; **Figure 4D**), and ECO30 WT likewise demonstrated enhanced invasion relative to both controls (P < 0.001 and P < 0.05; **Figure 4E**). Although ECO42 WT maintained higher invasion rates than ATCC25922 (P < 0.01) and ECO42Δ*eprX* (P < 0.05; **Figure 4F**), the difference was less marked than in the other strains. Collectively, these phenotypic measurements are consistent with the interpretation that loss of *eprX* is associated with, rather than definitively causative of, impaired host cell adhesion and invasion capacity, pending confirmation via genetic complementation.

### Gene expression profiling and differential expression analysis upon eprX deletion

**Figure 5:**
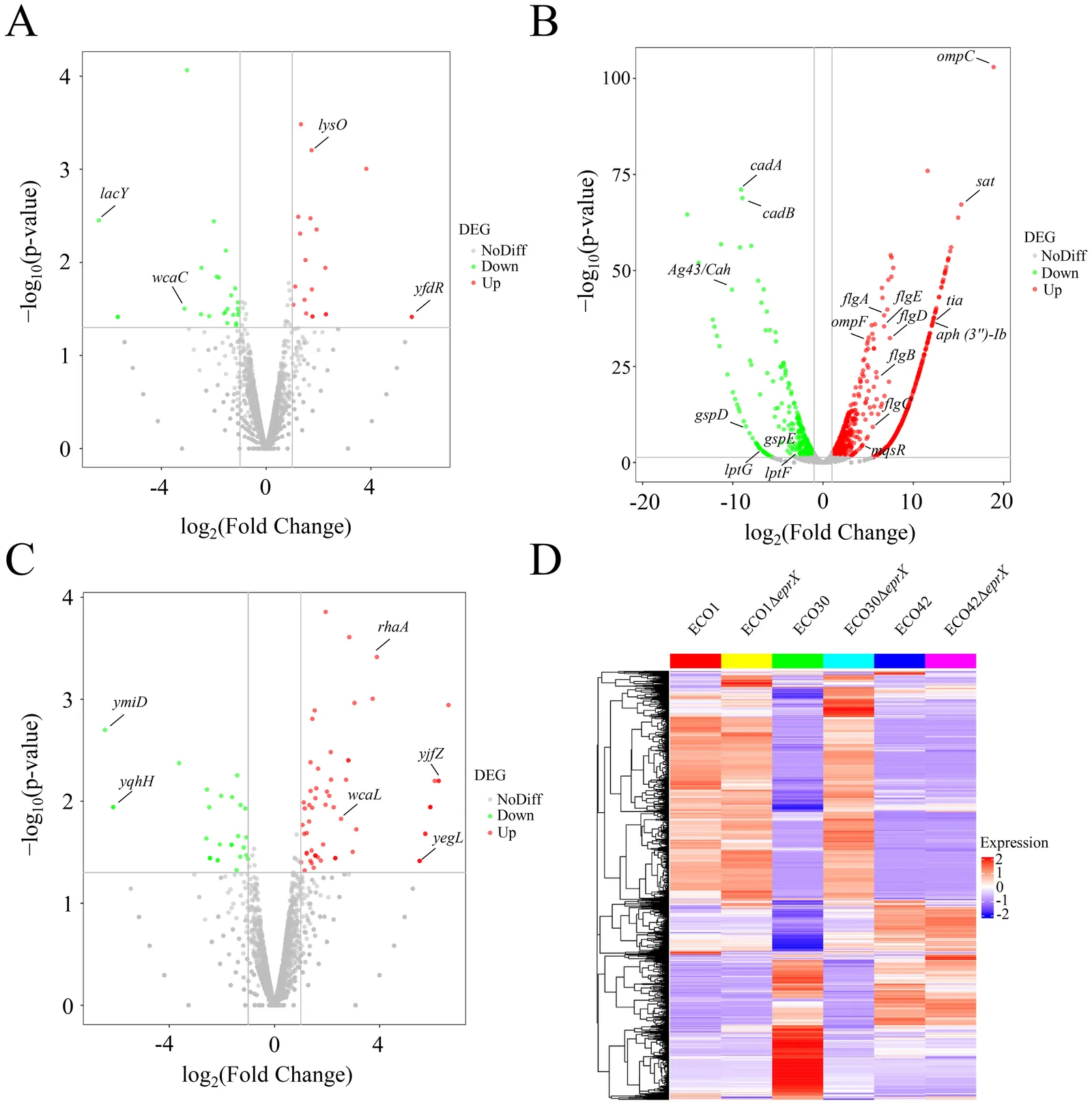
Transcriptomic analysis reveals global reprogramming upon *eprX* deletion. Volcano plots of DEGs for each strain: **(A)** ECO42Δ*eprX* vs ECO42, **(B)** ECO30Δ*eprX* vs ECO30, and **(C)** ECO1Δ*eprX* vs ECO1. In these plots, the x-axis represents log_2_(fold change) of gene expression and the y-axis represents statistical significance (−log_10_(*p*-value)). Genes with significantly altered expression are highlighted: red dots indicate upregulated genes in the Δ*eprX* strain relative to the wild-type (WT); green dots indicate downregulated genes; and gray dots represent genes with no significant differential expression. **(D)** Heatmap of gene expression levels (log_2_(fold change)) for DEGs across six samples from three *E. coli* strains (ECO1, ECO30, and ECO42) and their respective Δ*eprX* mutants. The color key (right-hand side) indicates the magnitude of expression: red hues represent higher expression levels, while blue hues represent lower levels. Hierarchical clustering (dendrograms on the left and top) was performed on the rows (genes) and columns (samples) to group genes and samples with similar expression profiles, respectively.

To examine the transcriptional changes accompanying *eprX* deletion in *E. coli* strains ECO1, ECO30, and ECO42, we performed comparative transcriptomics (**Figure 5D**; **Supplementary Tables S4**-**S6**).

RNA sequencing was used to quantify global gene expression in WT and Δ*eprX* strains. Deletion of *eprX* produced distinct transcriptional profiles across the three *E. coli* strains (**Supplementary Figure 4**; **Supplementary Table 7**). In the ECO42Δ*eprX* vs. ECO42 comparison, 45 differentially expressed genes (DEGs) were identified, comprising 20 upregulated and 25 downregulated genes (**Figure 5A**; **Supplementary Table 8**). In contrast, the ECO30 Δ*eprX* strain exhibited a far more extensive response, with 1,240 DEGs (828 up-, 412 down-regulated), indicating that the impact of *eprX* deletion on the transcriptome is highly strain-dependent (**Figure 5B**; **Supplementary Tables 8**, **S9**). In ECO1, 89 DEGs were identified (62 up-, 27 down-regulated) (**Figure 5C**; **Supplementary Table 10**). These transcriptional alterations coincided with the phenotypic shifts in antibiotic susceptibility and virulence observed in these mutants.

Regarding antibiotic resistance, the most notable finding was the marked upregulation of *ompC* (log_2_FC = 18.89, *P*adj < 4.78 × 10^-100^, which encodes a major outer membrane porin (**Figure 5B**; **Supplementary Table 9**). OmpC facilitates the permeability of aminoglycosides such as gentamicin (Chen et al., 2025). This increased expression is consistent with, and may contribute to, the observed hypersensitivity. While complementation is needed to confirm causality, these transcriptional changes are consistent with loss of EprX being associated with elevated porin transcript abundance. Another porin gene, *ompF*, was also significantly upregulated (log_2_FC = 4.96, *Padj* < 1.46 × 10⁻³^0^; **Supplementary Table 9**), supporting a potential link to heightened sensitivity to gentamicin and ciprofloxacin. The notable upregulation of *aph(3”)-Ib* (log_2_FC = 12.86, *Padj* < 8.70 × 10⁻^4^²) may represent a transcriptional response coincident with increased antibiotic influx via upregulated porins, although this response was insufficient to restore resistance phenotypes in the mutant background.

For virulence-associated changes, the serine protease autotransporter toxin *sat* was dramatically upregulated (log_2_FC = 14.97, *P*adj < 1.10 × 10^-61^), which may reflect dysregulated rather than physiologically controlled toxin production (**Figure 5B**; **Supplementary Table 9**). The invasion-associated outer membrane protein *tia* was also markedly upregulated (log_2_FC = 13.23, *P*adj < 1.66 × 10^-45^), consistent with a possible inappropriate activation of the invasion machinery (**Figure 5B**; **Supplementary Table 9**). Most notably, numerous flagellar genes—including *flgA*, *flgB*, *flgC*, *flgD*, *flgE*, and *fliP*—were strongly induced, suggesting potential misregulation that could impair the coordinated motility required for effective host cell invasion (**Figure 5B**; **Supplementary Table 9**). Elevated transcript levels of toxin–antitoxin system components (*hokD*, *mqsR*, *mqsA*) were observed and may reflect activation of generalized stress responses, which could indirectly affect virulence-associated pathways. Increased​ transcript levels of LPS biosynthesis genes (*waaO*, *rfaY*) were observed (**Figure 5B**; **Supplementary Table 9**). Given the role of LPS in envelope integrity, these changes might alter outer membrane composition, potentially affecting antibiotic permeability and host interactions. While these LPS-associated changes could modulate surface properties relevant to antibiotic sensitivity and virulence, direct causal relationships remain to be established through complementation analyses.

Analysis of downregulated DEGs in the ECO30 Δ*eprX* strain identified genes linked to both increased antibiotic sensitivity and reduced virulence. *eprX* deletion was associated with significant downregulation of 352 genes, including 96 implicated in antibiotic resistance and 17 linked to virulence factors (**Supplementary Table 9**).

The most notable findings include downregulation of LPS transport system components *lptG* (log_2_FC = −7.45, *P*adj < 5.41 × 10^-5^) and *lptF* (log_2_FC = −1.55, *P*adj < 2.79 × 10^-4^), which may correlate with compromised outer membrane integrity and reduce the barrier against antibiotics such as gentamicin and ciprofloxacin, while also potentially decreasing LPS production—a key virulence factor for endotoxicity and serum resistance (**Figure 5B**; **Supplementary Table 9**). These observations are consistent with the observed phenotypic changes, though causal links remain to be established via genetic complementation. Downregulation of *cadA* (log_2_FC = −9.08, *P*adj < 1.31 × 10^-68^) and *cadB* (log_2_FC = −8.93, *P*adj < 1.58 × 10^-68^), encoding cadaverine biosynthesis enzymes, may impair biofilm formation capacity and acid tolerance, potentially contributing to the increased antibiotic susceptibility and diminished virulence (**Figure 5B**; **Supplementary Table 9**). Reduced expression of Type II secretion system components (*gspD*, *gspE*, *gspF*) may correlate with impaired secretion of toxins and virulence factors, consistent with the trend toward reduced HeLa cell invasion, while also potentially limiting export of resistance-associated enzymes. Downregulation of the autotransporter adhesin *ag43* (log_2_FC = −6.83, *P*adj < 2.87 × 10^-40^) may further weaken host cell adhesion and biofilm formation, exacerbating both attenuated virulence and increased antibiotic sensitivity (since biofilms confer antimicrobial protection). Finally, reduced expression of multidrug efflux pump genes *macB*, *mdtC*, and *emrB* in *eprX*-deficient strains is correlated with diminished antibiotic extrusion capacity, as inferred from susceptibility phenotypes (**Figure 5B**; **Supplementary Table 9**).

### Functional enrichment of DEGs upon eprX deletion

Functional enrichment of DEGs in the *eprX*-knockout ECO30 strain is consistent with a potential role for *eprX* deletion in increased susceptibility to gentamicin and ciprofloxacin and attenuated virulence (as evidenced by decreased *G. mellonella* toxicity alongside reduced HeLa cell adhesion/invasion; **Supplementary Figure 5**; **Supplementary Tables S11**-**S13**).

**Figure 6:**
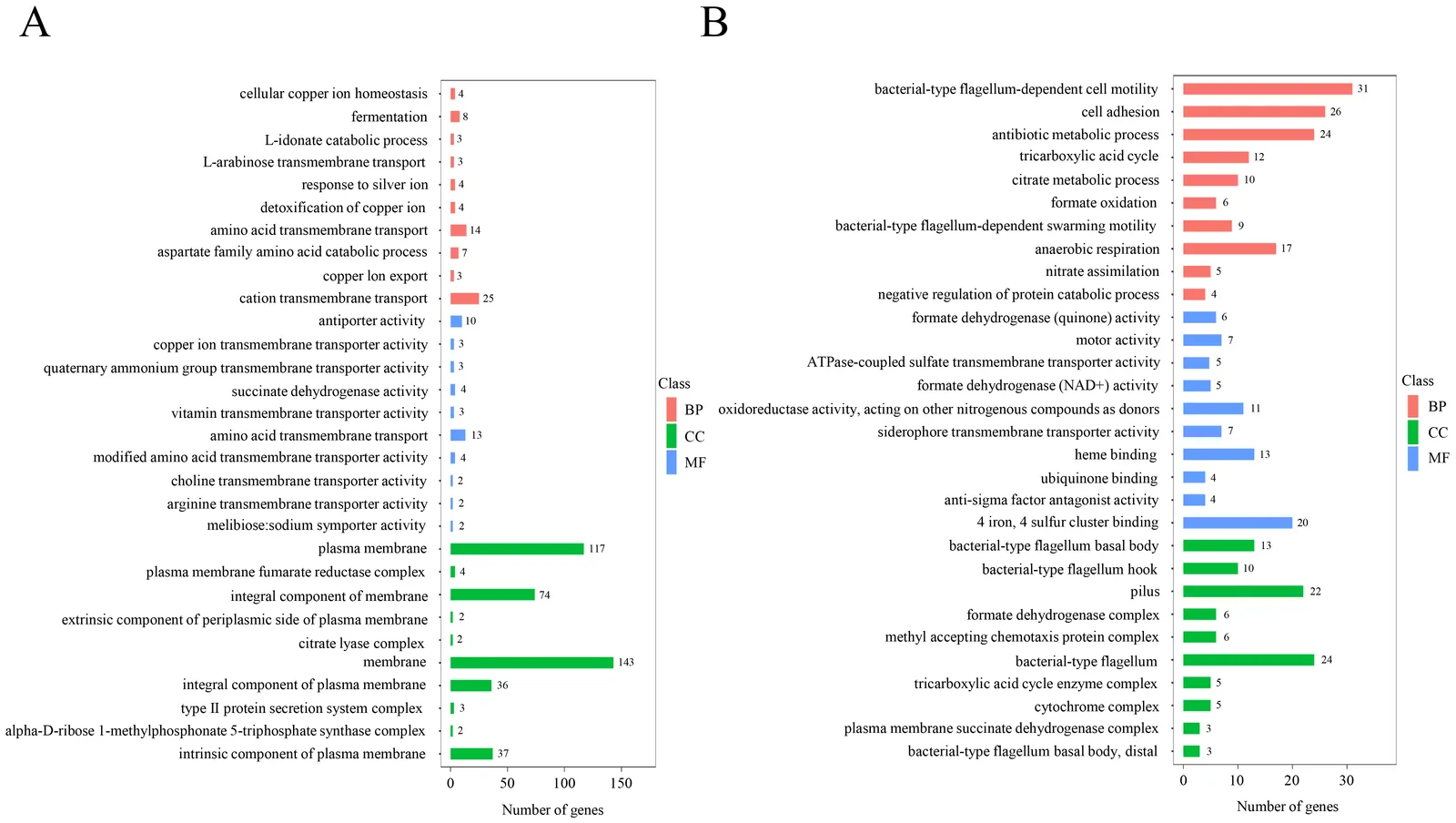
Functional enrichment of DEGs upon *eprX* deletion in ECO30 strain. **(A)** GO enrichment of down-regulated DEGs. **(B)** GO enrichment of up-regulated DEGs. In both panels, the y-axis lists GO terms categorized as BP (Biological Process, red), CC (Cellular Component, green), and MF (Molecular Function, blue).

Among these categories, several key functional clusters correlate with the observed phenotypic changes (**Figure 6**; **Supplementary Figure 5**). Downregulation of cation transmembrane transport (25 genes), including amino acid transport (14 genes) and antiporter activity (10 genes), could reflect perturbations in ion homeostasis and nutrient uptake under stress—changes that could contribute to antibiotic tolerance and intracellular survival during infection (**Figure 6A**). Similarly, the reduced enrichment of copper ion homeostasis and detoxification genes (e.g., export and detoxification) suggests a potential impairment in protective mechanisms against reactive oxygen species and metal stress, which are known to be involved in antibiotic-mediated killing and host immune evasion (Derke et al., 2020; Hyre et al., 2021). Downregulation of plasma membrane–associated components (e.g., integral membrane components, fumarate reductase complex) implies possible compromised membrane integrity, which could diminish​ efflux pump activity and modulate surface properties required for host cell adhesion (May and Grabowicz, 2018; Misra et al., 2015). Finally, the reduced representation of transporters for modified amino acids, choline, arginine, and quaternary ammonium groups may signify diminished metabolic adaptability and altered surface charge, potentially compromising bacterial fitness in hostile environments and affecting host-cell interactions (Ahmad et al., 2025; Iyer et al., 2003; Lamark et al., 1996).

Conversely, upregulated functional categories in the *eprX*-knockout *E. coli* ECO30 strain offer tentative mechanistic clues that may link transcriptional shifts to the enhanced antibiotic susceptibility and attenuated virulence (**Figure 6B**; **Supplementary Figure 5**). At the biological process level, significant enrichment of flagellum-dependent cell motility (31 genes), swarming motility (9 genes), and cell adhesion (26 genes) suggests dysregulation of flagellar assembly. Because proper flagellar function is critical for bacterial colonization, biofilm formation, and productive host-cell interaction—all established virulence determinants— this dysregulation likely contributes to, but does not solely explain, the observed virulence defects (**Figure 6B**). Upregulation of anaerobic respiration (17 genes) and nitrate assimilation (5 genes) was detected and may reflect metabolic reprogramming or altered redox status, changes that have been linked to antibiotic tolerance in other contexts. Likewise, enrichment of antibiotic metabolic processes (24 genes) and formate oxidation (6 genes) indicates a possible shift toward enhanced engagement with formate pathways, which could influence intracellular redox states or drug-efflux dynamics. At the molecular function level, enrichment of motor activity (7 genes), ATPase-coupled sulfate transport (5 genes), and oxidoreductase activity (11 genes) points to potentially elevated energy-dependent transport and redox enzyme activity, which may modify intracellular antibiotic concentrations or stress responses. Notably, enrichment of heme binding (13 genes) and iron–sulfur cluster binding (20 genes) suggests augmented cofactor utilization, which might impact electron transport chain function and, by extension, antibiotic susceptibility via respiratory modulation. At the cellular component level, enrichment of bacterial-type flagella (24 genes), pili (22 genes), and flagellar basal bodies (13 genes) confirms structural alterations in motility and adhesion machinery, likely impairing host colonization and virulence.

### Differentially expressed gene pathway enrichment analysis

KEGG pathway enrichment analysis of differentially expressed genes (DEGs) in the *eprX*-knockout *E. coli* ECO30 strain suggests putative mechanistic insights into the observed associations between *eprX* deletion and enhanced susceptibility to gentamicin and ciprofloxacin, as well as attenuated virulence (**Supplementary Figure 6**; **Supplementary Tables S14**-**S16**).

**Figure 7:**
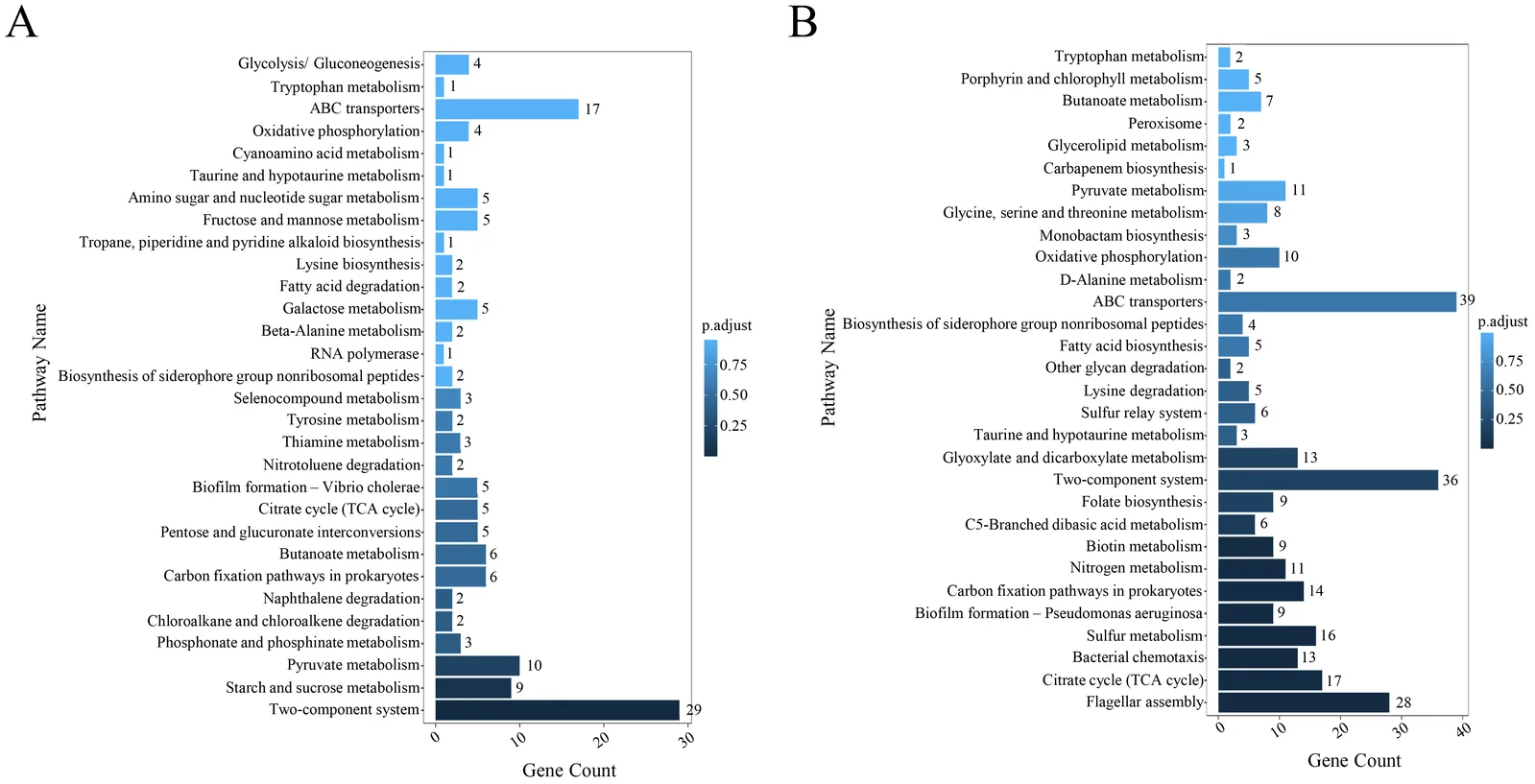
KEGG pathway enrichment analysis of DEGs in ECO30 strain​. **(A)** Downregulated genes; **(B)** Upregulated genes. Pathways are ranked by gene count (horizontal axis). Bar color indicates the adjusted *p*-value (p.adjust) using a blue gradient, where darker shades correspond to lower *p*-values and higher statistical significance.

The most prominently downregulated pathway was the two-component system (29 genes), which is critical for bacterial signal transduction, environmental sensing, and stress responses, including antibiotic resistance mechanisms such as efflux pump regulation and membrane permeability modulation (**Figure 7A**). The observed suppression of these pathways *may correlate* with altered adaptive responses to antimicrobial pressure, *potentially contributing* to the increased susceptibility phenotype. Similarly, ABC transporters (17 genes)—often involved in exporting antibiotics and maintaining cellular homeostasis under stress—were strongly downregulated. This reduction is consistent with the heightened drug sensitivity observed experimentally. Furthermore, downregulation of pyruvate metabolism (10 genes) and the TCA cycle (5 genes), both central to energy production and redox balance, could compromise bacterial fitness under antibiotic challenge and during host cell interaction. Other affected pathways included fatty acid degradation (2 genes), galactose metabolism (5 genes), and amino sugar/nucleotide sugar metabolism (5 genes), which contribute to envelope integrity and biosynthesis of virulence-associated structures. Their downregulation might weaken the bacterial cell surface and reduce the production of factors necessary for host colonization or immune evasion. Finally, the downregulation of biofilm formation pathways (e.g., *Vibrio cholerae* biofilm formation, 5 genes) and carbon fixation pathways (6 genes) implies impaired persistence and metabolic flexibility in hostile environments, further *potentially diminishing* virulence potential (**Figure 7A**).

Upregulated KEGG pathways in the *eprX*-knockout strain are consistent with the association with increased sensitivity to gentamicin and ciprofloxacin, as well as attenuated virulence traits, including reduced toxicity toward *Galleria mellonella* and diminished adhesion and invasion of HeLa cells (**Figure 7B**). Specifically, prominent upregulation of ABC transporter genes was observed and may represent a transcriptional response to altered membrane permeability or efflux capacity in the *eprX*-deficient background. Concurrently, strong enrichment of flagellar assembly (28 genes) raises the possibility of altered motility and chemotaxis machinery, which is often associated with host cell interactions and tissue colonization. Enrichment of the two-component system (36 genes) and carbon fixation pathways (14 genes) was detected, suggesting possible roles in signal transduction, metabolic adaptation, and the response to antibiotic pressure. Upregulated sulfur metabolism (16 genes) and bacterial chemotaxis (13 genes) may reflect, respectively, potential alterations in redox homeostasis and environmental sensing, which could influence bacterial survival and pathogenicity. Additionally, the observed enrichment of glyoxylate and dicarboxylate metabolism (13 genes) and oxidative phosphorylation (10 genes) may reflect metabolic shifts potentially underlying persistence or host adaptation. Differential expressions of nitrogen metabolism (11 genes) and phenylalanine, tyrosine, and tryptophan biosynthesis (8 genes) suggests possible alterations in amino acid and cofactor availability, which may influence toxin production or immune evasion.

## Discussion

We propose EprX, a YbjI-type pentapeptide repeat protein (PRP), as a candidate locus potentially involved in association with shifts in the resistance–virulence balance of clinical *E. coli* bloodstream isolates. EprX levels appear to correlate with baseline antibiotic resistance, whereas its deletion is further associated with upregulation of virulence determinants. Loss of *eprX* is therefore observed to coincide with divergent shifts between these traits, yielding a phenotype of heightened susceptibility and reduced virulence. Importantly, these findings reflect correlative associations rather than proven causality. Direct regulatory mechanisms remain to be established.

Specifically, the Δ*eprX* mutant exhibits markedly increased susceptibility to key antibiotics, including gentamicin and ciprofloxacin. This hyper-susceptibility is coincided with attenuated virulence in both *in vivo* and cell-based infection models. Together, these observations are consistent with a model in which *eprX* carriage is associated with a distinct adaptive state during systemic infection, although causal regulatory mechanisms remain to be defined.

The identification of EprX as a member of the YjbI family of PRPs merits attention. While PRPs have traditionally been characterized as cytoplasmic proteins that correlate with reduced susceptibility to DNA gyrase from quinolone antibiotics—exemplified by the Qnr proteins—emerging evidence indicates that YjbI-type PRPs can also localize to the cell envelope. There, they have been linked to altered outer membrane composition and modifications in antibiotic susceptibility (Collin et al., 2011; Costafrolaz et al., 2026). Structural modeling of EprX revealed the characteristic right-handed quadrilateral α-helical fold typical of PRPs, which is consistent with its classification within this family.

Among the 85 clinical *E. coli* bloodstream isolates analyzed, the *eprX* gene was detected in 21.2%, raising the possibility that this locus may represent a common yet previously underappreciated feature of circulating *E. coli* clones. Notably, the genomic context of *eprX* varies considerably, which may imply that the acquisition or loss of *eprX* could be mediated by mobile genetic elements, potentially correlating with accelerated adaptation of *E. coli* to clinical environments (Kanai et al., 2022; Larsson and Flach, 2022).

The antimicrobial susceptibility phenotype of ECO30 Δ*eprX* is broadly consistent with its transcriptomic alterations in outer membrane permeability. The major porins *ompC* and *ompF* are dramatically up-regulated. Because these porins mediate the passive diffusion of hydrophilic antibiotics (Choi and Lee, 2019; Pages et al., 2008), their overexpression is expected to increase cellular entry of aminoglycosides and fluoroquinolones, thereby potentially lowering the MIC (Choi and Lee, 2019; Nikaido, 2003). This is a plausible molecular explanation consistent with observed increases in susceptibility of *EprX*-deficient mutants to gentamicin and ciprofloxacin. In the absence of EprX, several multidrug efflux pumps (*macB*, *mdtC*, *emrB*) (Andersen et al., 2015; Di Maso and Ruiz, 2024) and components of the EnvZ/OmpR two-component regulatory system (Yuan et al., 2011) are concomitantly down-regulated. These expression shifts are likely to be associated with reduced transcript levels of efflux machinery, which might contribute to diminished antibiotic extrusion capacity (Novelli and Bolla, 2024). Although the aminoglycoside-modifying enzyme APH(3″)-Ib is compensatorily up-regulated, this response appears insufficient to counteract the enhanced antibiotic influx through hyper-expressed porins. Collectively, these data raise the possibility that *eprX* carriage may be linked to alteration in porin and efflux pump profiles, thereby affecting antimicrobial susceptibility to some extent.

Phenotypic assays suggest that eprX knockout is associated with attenuated virulence in the *Galleria mellonella* infection model. Larval survival at 60 h post-infection was only 40%–60% for the wild-type strains ECO1, ECO30 and ECO42, but rose to 65%–95% for their corresponding Δ*eprX* mutants. *in vitro* cellular assays reveal that Δ*eprX* mutants exhibit decreased HeLa cell adhesion and invasion relative to wild-type strains, consistent with a possible association between *eprX* presence and baseline virulence potential of bloodstream *E. coli*.

Integrated transcriptomic data show that *eprX* deficiency is correlated with broad shifts in the expression of multiple host-interaction pathways, consistent with a potential role for EprX in the observed transcriptional landscape of virulence factors. In Δ*eprX* mutants, transcriptomic profiling identified significant downregulation of genes encoding type II secretion system (T2SS) components (*gspD*, *gspE*, *gspF*) (Cianciotto and White, 2017; Korotkov et al., 2012) and the autotransporter adhesin Ag43 (Klemm et al., 2004). These expression changes are predicted to compromise toxin secretion and bacterial adhesion, consistent with established roles of T2SS and Ag43 in these processes, though direct causal links between EprX loss and the observed phenotypic outputs await validation via complementary genetic or biochemical assays. Given the established roles of *cadA*/*cadB* in biofilm formation and acid tolerance (Akhova et al., 2021; Du et al., 2021), their downregulation in Δ*eprX* mutants was consistent with a contribution of EprX to the regulation of these phenotypes. While this association supports a potential contribution of the *cad* operon to these EprX-dependent traits, we cannot rule out that additional co-regulated pathways also underlie the observed phenotypic defects. Concurrently, decreased expression of the LPS transport genes *lptG*/*lptF* (Ruiz et al., 2008) in Δ*eprX* mutants is associated with compromised outer membrane barrier integrity, which could plausibly increase antibiotic permeability and reduce serum resistance.

Some virulence factors are notably up-regulated in Δ*eprX*, including the Sat toxin (Guyer et al., 2002) and the Tia invasin (Mammarappallil and Elsinghorst, 2000). This up-regulation is accompanied by elevated expression of flagellar genes and stress-related toxin–antitoxin systems (*hokD*, *mqsR*) (Qiu et al., 2022). The shift from controlled to dysregulated expression appears to disrupt metabolic homeostasis and functional coordination, potentially culminating in an overall attenuation of pathogenicity.

Based on observed transcriptional and phenotypic profiles, we propose a speculative working model in which EprX may be analogized to a “voltage regulator” to facilitate the conceptualization of its potential involvement in global metabolic and transcriptional networks potentially associated with resistance and virulence trade-offs. Our transcriptomic data show that EprX carriage is associated with lower transcript levels of multiple virulence-associated determinants, raising the possibility of resource redistribution toward outer membrane homeostasis and basal antimicrobial tolerance. However, direct evidence for energy conservation or quantitative resource allocation (e.g., via metabolomics or flux analysis) is currently lacking. This proposed modulatory effect may be triggered or influenced by specific host signals, thereby facilitating the full pathogenic program. The differential transcriptomic responses across isolates further suggest that the impact of *eprX* deletion is highly context-dependent, varying substantially based on each strain’s native genomic background.

Transcriptomic profiling of the Δ*eprX E. coli* ECO30 strain reveals differential expression of genes involved in lipopolysaccharide (LPS) biosynthetic and transport pathways. These changes are correlated with and likely contribute to the observed shifts in antimicrobial susceptibility and virulence. We observed reduced transcription of the LPS transport genes *lptG* and *lptF*, which co-occurs with transcriptional signatures that may indicate altered outer membrane architecture. The outer membrane serves as the primary permeability barrier restricting entry of hydrophilic antibiotics such as gentamicin and ciprofloxacin (Harrison et al., 2019; Ruiz et al., 2008). Thus, our RNA-seq data raise the possibility that EprX regulation of *lptG*/*lptF* might influence antibiotic susceptibility, though this link remains correlative at present. Concurrently, the bacterium upregulates aminoglycoside-modifying enzymes such as APH(3”)-Ib, suggesting a potential compensatory mechanism that could neutralize intracellular antibiotic accumulation, which is often associated with enhanced susceptibility. Reduced LPS export and assembly further is correlated with reduced density of surface-exposed O-antigen and core-oligosaccharide moieties, which are critical for resisting complement-mediated serum killing and dampening innate immune recognition (Krzyzewska-Dudek et al., 2024; Raetz and Whitfield, 2002; Whitfield and Trent, 2014). Loss of this protective layer is associated with increased display of underlying pathogen-associated molecular patterns (PAMPs) and core epitopes, rendering the mutant more vulnerable to the antimicrobial peptide- and hemocyte-mediated clearance arsenal of the *G. mellonella* hemocoel (Kavanagh and Reeves, 2004; Pereira et al., 2018; Tsai et al., 2016), which could account for its attenuated *in vivo* virulence. Further supporting this link, the transcriptome captured significant upregulation of LPS biosynthesis genes, including *waaO* and *rfaY*, in the *eprX* knockout background. While this initially appears contradictory, it is correlated with the broader pattern of transcriptional dyshomeostasis associated with loss of EprX. Rather than restoring balanced LPS assembly, these misregulated biosynthetic pathways may disrupt the stoichiometric coordination of lipid A, core, and O-antigen synthesis, which could potentially generate structurally aberrant LPS species that may fail to properly integrate into the outer membrane. This structural disorganization may contribute to increased membrane permeability and alter surface physicochemical properties, potentially compromising interactions with host epithelial cells and thereby diminishing both adhesion and invasion capacities. These LPS-associated transcriptional changes suggest a potential role for *eprX* in modulating outer membrane composition, though direct regulatory interactions require biochemical validation.

Our transcriptomic data are consistent with a model in which EprX may participate in metabolic and transcriptional programs potentially linked to *E. coli* fitness and a potential trade-off between antimicrobial resistance and virulence. Despite being based on correlative data, these observations require further validation to establish direct regulatory mechanisms. Upon *eprX* deletion, the most striking metabolic shift is the downregulation of the two-component system (TCS) pathway. TCS is a predominant signal-transduction architecture through which bacteria sense and integrate environmental cues to reconfigure adaptive responses (Stephenson and Hoch, 2002; Stock et al., 2000). This is consistent with the established role of the DegS–DegU two-component circuit as the primary gatekeeper of extracellular protease expression and associated transition-phase physiology in firmicutes (Mader et al., 2002; Msadek et al., 1990). This associated transcriptional downregulation of two-component signaling pathways may be linked to a reduced ability to induce efflux pump expression, remodel outer membrane permeability, and activate stress response programs under antibiotic pressure, which could collectively contribute to increased susceptibility to gentamicin and ciprofloxacin. Concurrently, the downregulation of ABC transporter pathways is associated with reduced export of antimicrobial compounds, while suppression of pyruvate metabolism and TCA cycle activity is concomitant with diminished energy production and redox homeostasis. These metabolic alterations may correspond to reduced metabolic flexibility, potentially affecting bacterial survival under antibiotic pressure and during host colonization. These energy deficits also could cascade into impaired envelope integrity, as reflected by the downregulation of fatty acid degradation, galactose metabolism, and amino sugar/nucleotide sugar metabolism pathways, which may disrupt lipopolysaccharide (LPS) biosynthesis and outer membrane stability. Specifically, reduced LPS production would be expected to lower the barrier against antibiotic entry and may attenuate endotoxic activity and serum resistance, providing a potential link between metabolic perturbation and both increased drug sensitivity and diminished virulence.

Complementary upregulated pathways are consistent with a model in which EprX loss may contribute to maladaptive metabolic rewiring that could elevate antibiotic susceptibility while concurrently potentially reducing both *in vitro* adhesion/invasion and *in vivo* virulence. The apparent upregulation of flagellar assembly and bacterial chemotaxis pathways likely reflects hyperactivation of the motility machinery. This response correlates with, and may contribute to, uncoordinated host–cell interactions and tissue colonization (Akahoshi and Bevins, 2022; Lovewell et al., 2011; Sevrin et al., 2020), and parallels the attenuated lethality observed in the *Galleria mellonella* model—an outcome broadly consistent with diminished adhesion and invasion in cellular assays. Upregulated ABC transporter pathways here may represent a potential, albeit ineffective, compensatory response to impaired efflux capacity, which could further correlate with reduced capacity to expel aminoglycosides and fluoroquinolones. Metabolic reprogramming toward sulfur metabolism, glyoxylate and dicarboxylate metabolism, and oxidative phosphorylation likely alters intracellular redox states and energy allocation (Husain et al., 2010; McKinney et al., 2000; Puckett et al., 2017; Shatalin et al., 2011), potentially diverting resources away from virulence factor production and toward stress survival (Bergman et al., 2024; Dalebroux and Swanson, 2012; Rohmer et al., 2011). This shift is compatible with the dysregulated expression of toxin, autotransporter, and secretion system components observed in the transcriptome. Notably, the concurrent upregulation of amino acid biosynthesis pathways (phenylalanine, tyrosine, tryptophan) and nitrogen metabolism may signal altered cofactor availability that could underlie disrupted regulated expression of secreted toxins and adhesins, thus possibly contributing to the coupled reduction of adhesion/invasion phenotypes and pathogenicity (Amon et al., 2010; Caballero-Flores et al., 2020; Reitzer, 2003). Collectively, our correlative data are consistent with a tentative model in which EprX might constrain transcriptional investment in metabolically costly virulence pathways, potentially favoring homeostasis of envelope integrity, efflux capacity, and energy metabolism that could contribute to antibiotic tolerance. Crucially, this model remains speculative and requires experimental validation.

While our study focuses on the molecular mechanisms linking EprX to resistance and virulence, the epidemiological features of *eprX* carriage in our cohort merit consideration regarding their public health implications. The 21.2% prevalence of *eprX* among bloodstream *E. coli* isolates is compatible with the possibility that this locus represents a common, yet previously unrecognized, component of the accessory genome of clinical *E. coli*. The mobile genetic context of *eprX* (variable insertion between *pgpA* and *yaiO*) raises the possibility that it may be readily disseminated across strains, potentially facilitating the spread of the resistance-virulence regulatory network we describe here. The three distinct EprX sequence variants we identified offer a basis for future molecular epidemiology studies: tracking the distribution of these variants across geographic regions, clinical settings, and patient populations will be required to clarify whether specific *eprX* alleles correlate with hypervirulent or multidrug-resistant clones.

We acknowledge that the current study lacks comprehensive epidemiological annotation of our isolate cohort. Because the collection was retrospective and anonymized (as stipulated by our ethical approval), we were unable to formally assess correlate *eprX* carriage with patient demographics, infection sources, ESBL/carbapenemase production, sequence types, phylogroups, or clinical outcomes. Addressing these gaps remains a priority in our ongoing work. We are assembling an expanded cohort of *eprX*-positive bloodstream infection (BSI) isolates with fully annotated clinical metadata. This will enable systematic analysis of associations between *eprX* carriage and key clinical endpoints, including 30-day mortality, length of hospitalization, and recurrence of bacteremia. These data will be essential to attempt to translate our mechanistic findings into actionable insights for clinical microbiology and infection control.

Our findings also suggest broader ecological and evolutionary implications of the resistance–virulence trade-off. The “cost of resistance” is a well-documented phenomenon whereby expressing resistance determinants imposes a fitness burden on bacteria in the absence of antibiotics (Andersson and Hughes, 2010; Vogwill and MacLean, 2015). Given the apparent association between resistance maintenance (via porin/efflux control) and virulence suppression, EprX might represent a strategy that could potentially aid *E. coli* in conserving energy and resources during asymptomatic colonization or bloodstream transit. Under this model, the bacterium might commit to a high-virulence, high-metabolic-cost state when reaching a permissive niche (e.g., the kidney or meninges). This potential strategic plasticity is a candidate mechanism that may account for why some bloodstream isolates appear to retain high virulence potential despite being susceptible to multiple antibiotics *in vitro*.

The *G. mellonella* model has proven to be a valuable tool for capturing this nuanced *in vivo* behavior, enabling high-throughput virulence assessment while avoiding the ethical and logistical constraints of mammalian experimentation. However, as an invertebrate host, it lacks adaptive immune components (e.g., T and B cells) and differs from mammals in temperature regulation and metabolic physiology, which may limit direct translation to human infection. Future validation in mammalian models (e.g., murine sepsis models) is therefore warranted to assess the clinical relevance of EprX in human pathophysiology.

The stark differences in the magnitude of transcriptional reprogramming across the three clinical isolates—with 1,240 DEGs in ECO30, 89 in ECO1, and only 45 in ECO42—underscore the context-dependent nature of EprX-associated regulatory effects in genetically heterogeneous pathogens. Although all Δ*eprX* mutants exhibited consistent core phenotypes (heightened antibiotic susceptibility, attenuated virulence, and unchanged baseline growth), the vastly different scales of transcriptomic response show an association with several non-mutually exclusive features of their native genetic backgrounds that may contribute to variable transcriptional shifts after *eprX* deletion.

First, the three isolates harbor distinct EprX sequence variants (**Figure 1D**) and likely belong to divergent phylogenetic lineages with fundamentally different global regulatory architectures. Allelic variation in master transcriptional regulators (e.g., *hns*, *crp*, *arcA*) or sigma factors may influence EprX binding affinity for its targets or modify basal expression of downstream pathways, suggesting that such variation might potentially expand or contract the regulon associated with *eprX* loss.

Second, strain-specific carriage of mobile genetic elements—including plasmids, prophages, and insertion sequences—may introduce accessory regulators that are associated with EprX or relate to the accessibility of its target loci. For instance, ECO30 may carry a plasmid-encoded regulator that cooperates with EprX to repress porin expression. Deleting *eprX* would then derepress *ompC*/*ompF* and potentially trigger widespread transcriptional dysregulation. This interpretation is consistent with the near-total suppression of the two-component system (TCS) pathway observed uniquely in ECO30 (**Figure 7A**), which is consistent with downstream dysregulation. In contrast, ECO1 and ECO42 retained residual TCS activity that may be associated with cellular homeostasis.

Third, the degree of redundancy in native regulatory networks likely influences the impact of *eprX* deletion. ECO1 and ECO42 probably retain functionally overlapping regulators that appear to buffer against transcriptional shifts from EprX loss, which may in turn moderate downstream transcriptional responses. In contrast, ECO30 appears to lack such backup systems, such that *eprX* deletion leads to a near-collapse of regulatory homeostasis, which is reflected in the exceptionally high DEG count observed. Finally, pre-existing compensatory mutations or epistatic interactions unique to each isolate may decouple EprX from subsets of its regulon, which may limit the scope of transcriptional reprogramming in ECO1 and ECO42.

Importantly, the consistent core phenotypic shifts across all three mutant backgrounds indicate that *eprX* presence is associated with concerted shifts in resistance and virulence phenotypes across distinct clinical *E. coli* lineages, even though the breadth of its regulatory network varies across genetic backgrounds. To further dissect the genetic determinants of this strain-specific variability, we are performing comparative whole-genome sequencing of all 18 *eprX*-positive clinical isolates in our ongoing follow-up studies. These analyses should help clarify the extent to which lineage-specific polymorphisms, mobile element content, or regulatory network architecture are associated with these transcriptomic differences.

This study is subject to several limitations regarding clinical translation, mechanistic validation, and causal inference. Most importantly, the absence of genetic complementation precludes definitive attribution of the observed phenotypes to *eprX* deletion. Although our λ-Red knockout strategy was designed to minimize polar effects by preserving the flanking *pgpA* and *yaiO* loci, and the consistent phenotypic convergence across three genetically distinct clinical isolates suggests a reduced likelihood of spurious secondary mutations, restoring wild-type *eprX* in the mutant background remains the gold standard for establishing genotype–phenotype relationships. Without complementation, all conclusions from this study must be interpreted as associations rather than proven causal relationships. We plan to construct complemented strains—by reintroducing wild-type *eprX* into the Δ*eprX* background—to rigorously test whether the reported resistance, virulence, and transcriptional alterations are due to *eprX* loss.

Furthermore, the molecular mechanism underlying EprX function remains uncharacterized. Although transcriptomic profiling reveals global transcriptional changes upon *eprX* deletion, we lack direct biochemical evidence (such as DNA-binding assays, ChIP, or protein interaction mapping) to confirm whether EprX acts as a classical transcription factor, a structural scaffold, or an indirect modulator of envelope physiology. Consequently, the reported phenotypes and transcriptomic shifts represent correlative observations, and the underlying mechanism remains to be established. These transcriptional shifts could arise through three non-exclusive mechanisms: direct EprX binding to gene promoters, indirect modulation of global two-component regulatory systems, or downstream secondary stress responses triggered by disrupted membrane homeostasis and metabolic flux. Future biochemical studies using purified EprX will be essential to delineate its precise mode of action.

Additionally, we acknowledge that the RNA-seq findings presented here were not validated by RT–qPCR for representative DEGs (e.g., *ompC*, *ompF*, *macB*, *emrB*, *mdtC*, *lptF*, *lptG*, *gspD*, *ag43*). Although our RNA-seq data were generated from three biological replicates per strain and passed rigorous quality controls (Q30 >92%, alignment rate >96%), targeted RT–qPCR confirmation would provide additional quantitative rigor. We have therefore prioritized RT–qPCR validation as an essential next step in our ongoing work to strengthen the transcriptomic findings.

Looking ahead, subsequent work will aim to validate the regulatory role of EprX in mammalian infection models to assess its relevance to human pathophysiology. We will establish a murine intravenous sepsis model to evaluate the effects of *eprX* deletion on bacterial burden in blood and visceral organs (e.g., kidneys, liver), histopathological tissue damage, host cytokine responses, and survival outcomes. Should EprX be confirmed as a critical fitness node in systemic *E. coli* infection, targeted inhibition of its function—without relying on direct bactericidal agents—may represent a strategy to reduce virulence of multidrug-resistant strains, which could ultimately contribute to mitigating infection-related tissue damage. This approach warrants further investigation as a potential avenue for addressing the growing global threat of antimicrobial-resistant bacterial infections.

## Conclusion

EprX is a YjbI-type pentapeptide repeat protein whose detection in clinical bloodstream *E. coli* isolates is associated with broad transcriptional and phenotypic shifts. Integrated phenotypic and transcriptomic analyses are consistent with the hypothesis that *eprX* carriage might coincide with altered antimicrobial susceptibility profiles, as loss of the locus was observed to correlate with changes in outer membrane porin and multidrug efflux component transcript levels. Transcriptomic profiling further reveals an association between *eprX* positivity and comparatively lower transcript abundance of several virulence-associated genes under standard culture conditions. Collectively, our observations suggest that *eprX* represents a previously unreported genetic locus that correlates with simultaneous shifts in both antimicrobial susceptibility and virulence-related phenotypes across clinical *E. coli* isolates. We propose a hypothetical, correlative framework built on these phenotypic and transcriptional associations. Crucially, the findings presented herein remain strictly observational. In the absence of genetic complementation experiments, direct causal links between the *eprX* locus and the measured transcriptional and phenotypic changes remain to be established. Follow-up work—including construction and analysis of complemented strains, biochemical characterization of protein function, and mammalian sepsis infection models—will be necessary to test this model and investigate the potential mechanisms underlying the observed associations between EprX-associated molecular profiles and bacterial fitness in systemic infection.

## Data Availability

The datasets presented in this study can be found in online repositories. The names of the repository/repositories and accession number(s) can be found in the article/**Supplementary Material**.
