# Supplementary material for: EprX associates with concurrent shifts in antimicrobial resistance and virulence in clinical bloodstream E. coli: a putative adaptive node for bacterial fitness
Source: Front Cell Infect Microbiol. 2026 Aug 26;16:1919001. doi: 10.3389/fcimb.2026.1919001 (PMC13553396; doi:10.3389/fcimb.2026.1919001)
Supplement: Supplementary file 1 [file DataSheet1.pdf]

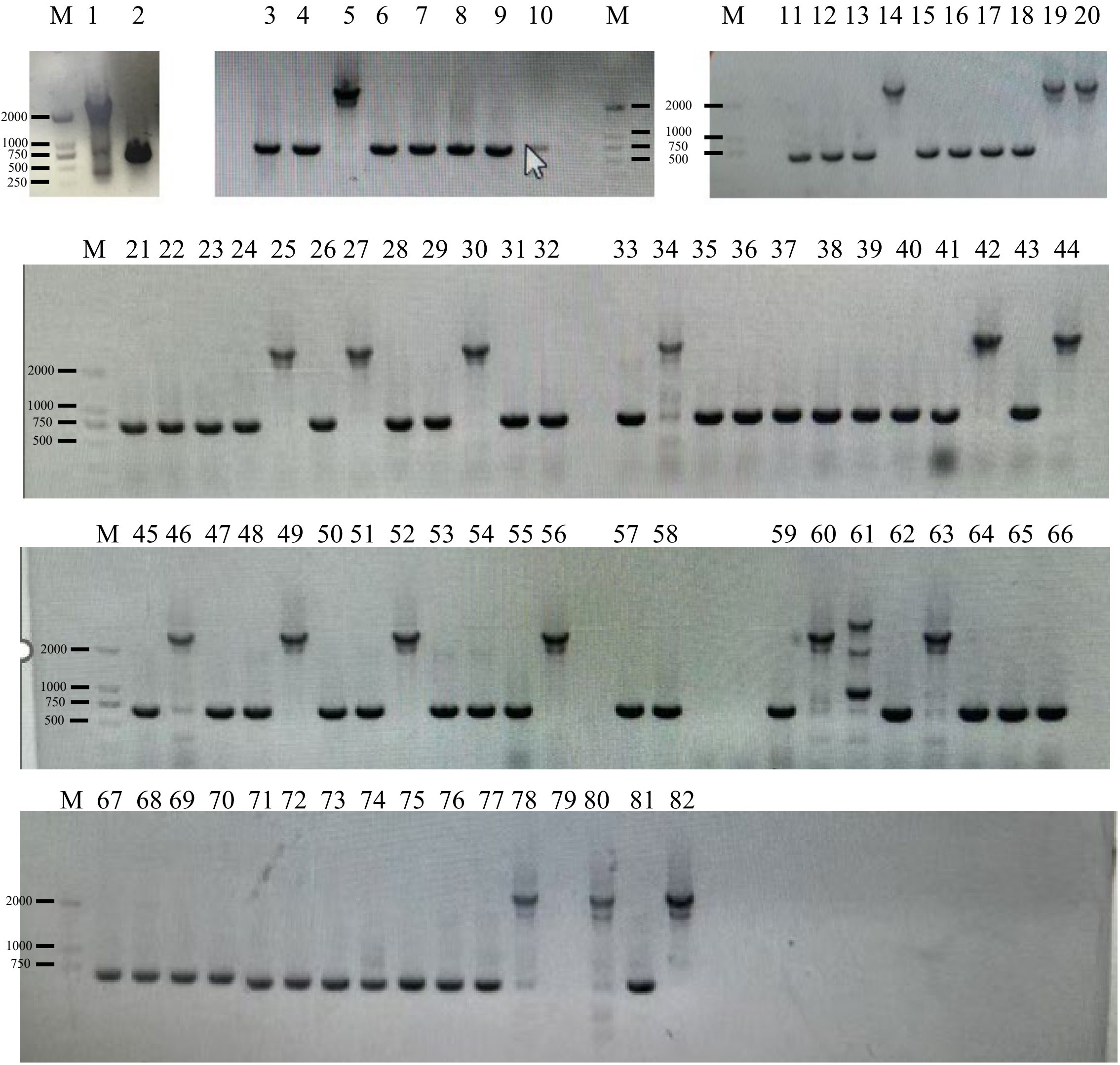
M 1 2
3 4 5 6 7 8 9 10 M
M 11 12 13 14 15 16 17 18 19 20
2000
1000
750
500
250
M 21 22 23 24 25 26 27 28 29 30 31 32 33 34 35 36 37 38 39 40 41 42 43 44
M 45 46 47 48 49 50 51 52 53 54 55 56 57 58 59 60 61 62 63 64 65 66
M 67 68 69 70 71 72 73 74 75 76 77 78 79 80 81 82
